# 4-hydroxyphenylpyruvate dioxygenase promotes lung cancer growth via pentose phosphate pathway (PPP) flux mediated by LKB1-AMPK/HDAC10/G6PD axis

**DOI:** 10.1038/s41419-019-1756-1

**Published:** 2019-07-08

**Authors:** Changliang Shan, Zhaoliang Lu, Zhen Li, Hao Sheng, Jun Fan, Qi Qi, Shuangping Liu, Shuai Zhang

**Affiliations:** 1 State Key Laboratory of Medicinal Chemical Biology, College of Pharmacy and Tianjin Key Laboratory of Molecular Drug Research, Nankai University, 300350 Tianjin, China; 2 The First Affiliated Hospital, Biomedical Translational Research Institute, Jinan University, 510632 Guangzhou, Guangdong China; 3 Department of Clinical Biological Resource Bank, Guangzhou Institute of Pediatrics, Guangzhou Women and Children’s Medical Center, Guangzhou Medical University, 510623 Guangzhou, Guangdong China; 4 Department of Medical Biochemistry and Molecular Biology, School of Medicine, Jinan University, 510632 Guangzhou, Guangdong China; 5 Department of Pharmacology, School of Medicine, Jinan University, 510632 Guangzhou, Guangdong China; 6 Department of Pathology, Medical School, Dalian University, 116622 Dalian, Liaoning China; 7 School of Integrative Medicine, Tianjin University of Traditional Chinese Medicine, 301617 Tianjin, China

**Keywords:** Cancer metabolism, Growth factor signalling, Gene silencing

## Abstract

4-hydroxyphenylpyruvate dioxygenase (HPD) is an important modifier of tyrosine metabolism. However, the precise contribution of HPD to cancer metabolism and tumorigenesis remains unclear. In this study, we found that HPD was highly expressed in lung cancer and its higher expression correlated with poor prognosis in lung cancer patients. Suppressed HPD expression was sufficient to decrease oxidative pentose phosphate pathway (PPP) flux, leading to reduced RNA biosynthesis and enhanced reactive oxygen species (ROS) level, attenuated cancer cell proliferation, and tumor growth. Mechanistically, HPD not only promotes tyrosine catabolism leading to increased acetyl-CoA levels, the source of histone acetylation, but also stimulates histone deacetylase 10 (HDAC10) translocation from the nucleus into the cytoplasm mediated by tumor suppressor liver kinase B1 (LKB1)–AMP-activated protein kinase (AMPK) signaling. Both controlled histone acetylation modification, which enhanced transcription of the important PPP enzyme Glucose-6-Phosphate Dehydrogenase (G6PD). Thus, this study reveals HPD as a novel regulator of LKB1–AMPK signaling-mediated HDAC10 nuclear location, which contributes to G6PD expression in promoting tumor growth, which is a promising target for lung cancer treatment.

## Introduction

Lung cancer is the leading cause of cancer mortality, which ranks as the first cancer-related mortality worldwide. Because of its aggressiveness, few symptoms during early disease stages, and metastasis to other organs before diagnosis, there is a critical need to elucidate the underlying molecular mechanisms and identify molecular targets in lung cancer.

Reprogramming of energy metabolism has been recognized as an emerging hallmark of cancer. Recently, studies report that metabolic enzymes play an important role in cancer cell metabolism and tumorigenesis^1,2^. The fact that the metabolism of tumor cells is altered has been known for many years. However, the mechanisms and consequences of metabolic reprogramming have just begun to be understood. Besides glucose anaerobic metabolism (known as Warburg effect), emerging evidence indicates that metabolism of other nutrients, such as glutamine, fatty acids, cholesterol, amino acids, and one carbon, is important for tumor proliferation^3–9^. Therefore, identifying the roles of metabolic enzymes involved in nutrient metabolism is required in order to uncover fundamental molecular events of malignancy and reveal new targets for cancer diagnosis and treatment.

Tyrosine is one of the 20 common amino acids found in all biological systems. It is catalyzed to fumarate and acetyl coenzyme A (acetyl-CoA)^10,11^. Fumarate enters into the citric acid cycle to be oxidized for energy production. Acetyl-CoA participates in many biochemical reactions in protein, carbohydrate and lipid metabolism, and energy production, supporting cell growth and proliferation^12,13^. In addition, acetyl-CoA is involved in the regulation of protein acetylation modification, histone modification, and gene expression^14^. 4-hydroxyphenylpyruvate dioxygenase (HPD) is a key enzyme involved in tyrosine catabolism, which catalyzes the conversion of 4-hydroxyphenylpyruvate into homogentisate^15,16^. Congenital HPD deficiency is a rare, relatively benign condition known as hereditary type III tyrosinemia^17,18^. However, the precise contribution of HPD to cancer metabolism and tumorigenesis is still largely unknown.

In this study, we demonstrated that the HPD–glucose-6-Phosphate Dehydrogenase (G6PD) axis enhanced the oxidative pentose phosphate pathway (PPP) flux and facilitated lung cancer growth. Further mechanistic studies showed that HPD was a novel regulator of tumor suppressor liver kinase B1 (LKB1)–AMP-activated protein kinase (AMPK) signaling-mediated histone deacetylase 10 (HDAC10) nuclear location and contributed to the G6PD expression. Moreover, we unveiled a previously unappreciated relationship between tyrosine metabolism pathway and anabolic biosynthesis mediated by HPD–Histone–G6PD axis, which offers a novel target for clinical application.

## Results

### HPD promotes cell proliferation and tumor growth in vitro and in vivo

**Fig. 1:**
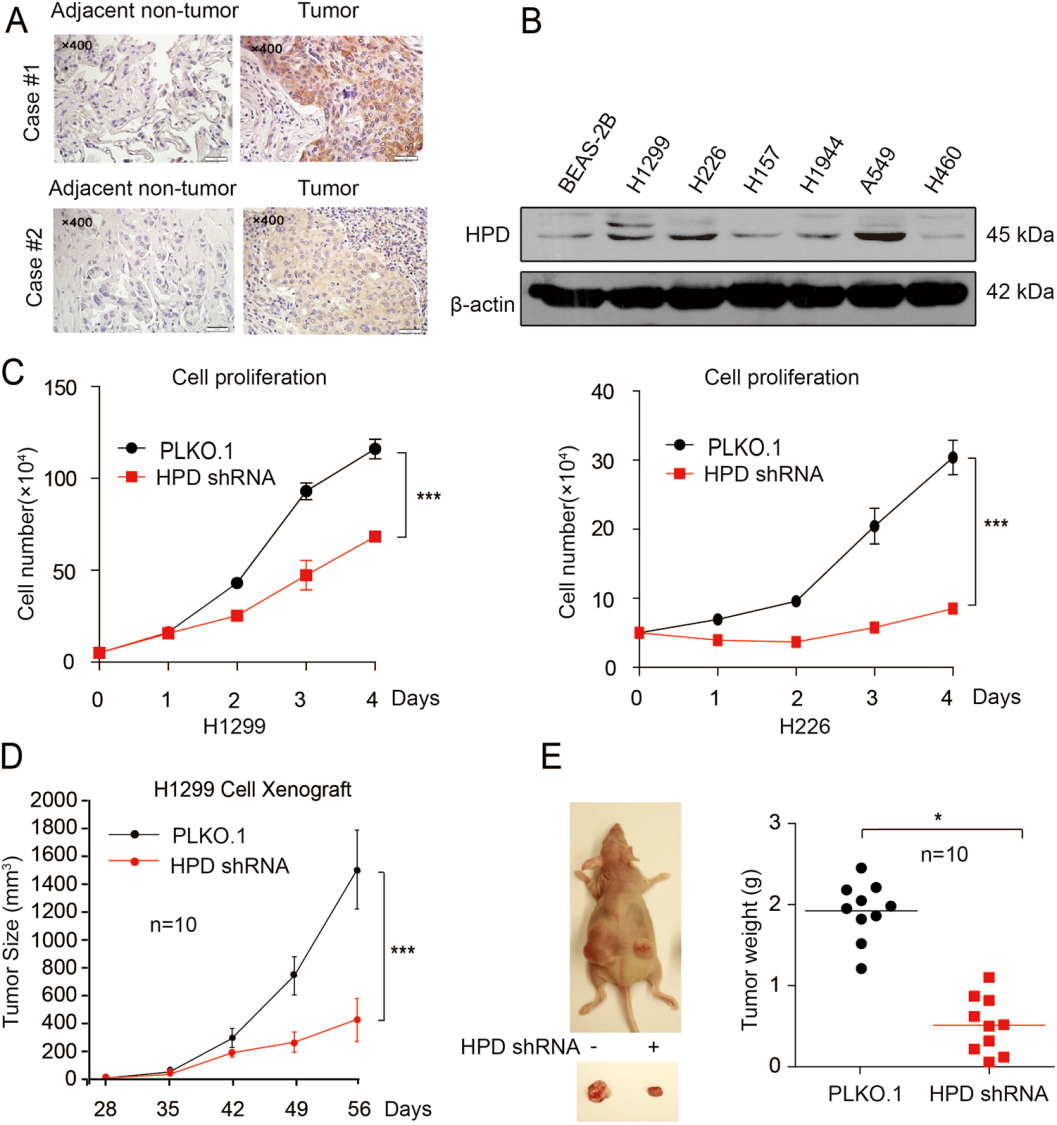
HPD expression is evaluated in lung cancer and is important for cancer cell proliferation and tumor growth. **a** HPD protein levels were analyzed in human tissue microarrays of 48 lung cancer tissues and 48 adjacent nontumor lung tissues by IHC staining. **b** HPD protein levels were analyzed in the majority of a spectrum of diverse human lung cancer cells, including H1944, H460, H1299, H157, H226 cells, and normal proliferating human bronchial epithelial cell line (BEAS-2B). **c** Cell proliferation rates determined by cell counting in human lung cancer H1299 and H226 cells with stable knockdown of HPD. **d** Tumor growth was compared between xenograft nude mice injected with HPD-knockdown H1299 cells and control vector cells. The values were given as mean ± SD. **e** *Left*: dissected tumors in a representative nude mouse are shown. *Right:* tumor mass in xenograft nude mice injected with HPD-knockdown H1299 cells compared with mice (*n* = 10) injected with the control vector cells

To determine the role of HPD in lung cancer, we first examined the expression of HPD in tissues from lung cancer patients. Immunohistochemistry (IHC) staining showed that the expression of HPD was positive in 40 (83.3%) and strongly positive in 30 (62.5%) of the 48 lung cancer patients, which was significantly higher than in adjacent nontumor lung tissues (Table S1) (Fig. 1a). No correlation was observed between tumor grade and HPD levels (Table S2). Moreover, higher levels of HPD are correlated with reduced overall survival based on the publicly available Kaplan–Meier Plotter (http://kmplot.com) (HPD: accession number 206024_at) (Fig. S1A). A panel of lung cancer cell lines also showed higher expression of HPD compared with normal human bronchial epithelial cell line (BEAS-2B) (Fig. 1b). All these findings indicate the potential role of HPD in tumor formation and progression.

To address the functional consequence of HPD upregulation in lung cancer, we examined the effect of HPD on cell proliferation and tumor growth. We established various HPD stable knockdown cell lines, and we found that depleting HPD downregulated cell proliferation of lung cancer cells (Fig. 1c). Nevertheless, interference with HPD in BEAS-2B cells did not affect their proliferation (Fig. S1B, C). Moreover, the oncogenic action of HPD was also confirmed by a xenograft model. As indicated in Fig. 1d, e, H1299 cells with HPD deficiency showed a slower growth rate compared with control cells. Collectively, these data indicate that HPD promotes lung cancer cell proliferation and tumor growth in vitro and in vivo, and correlates with overall patient survival.

### HPD reprograms oxidative PPP

**Fig. 2:**
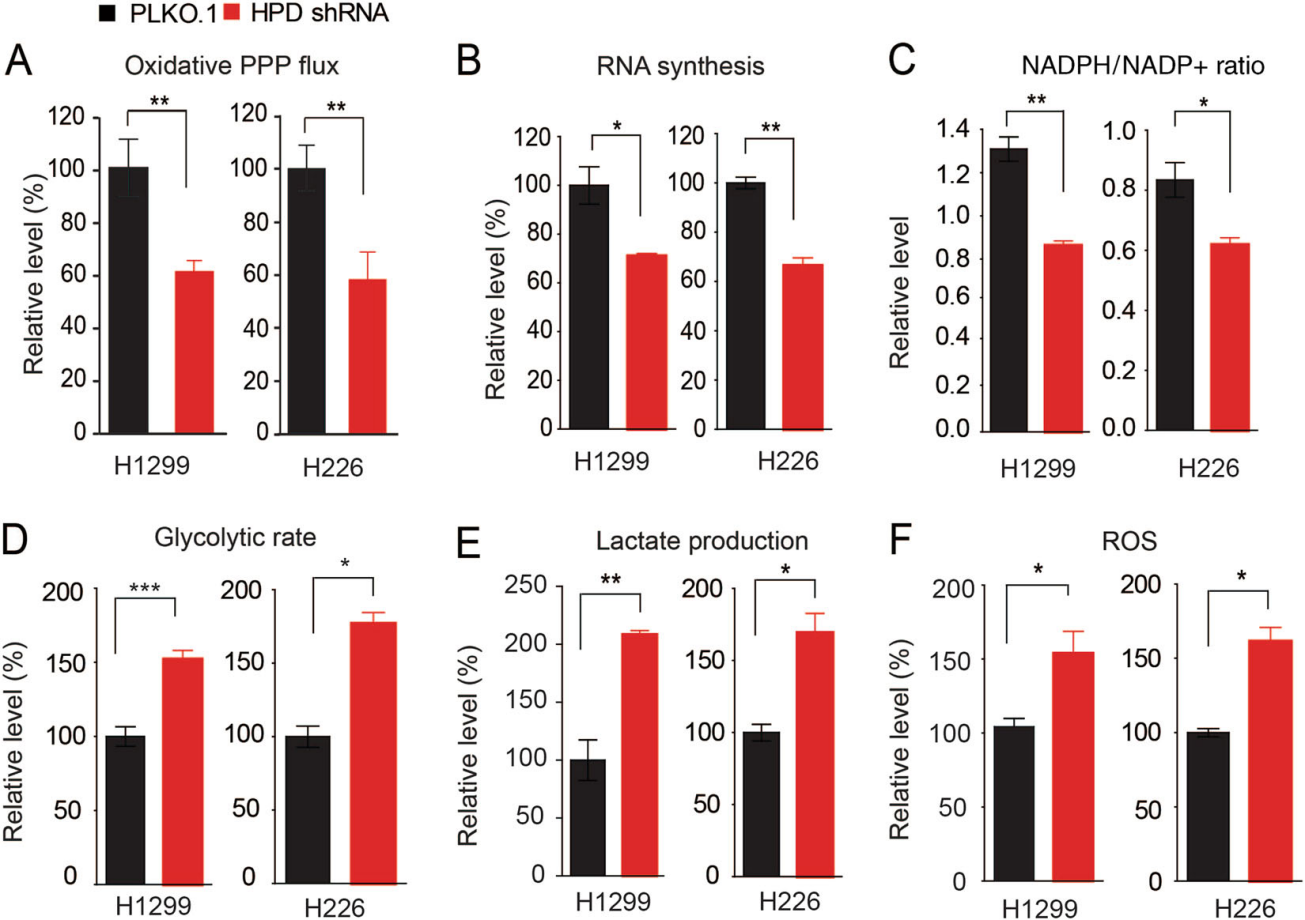
HPD reprograms oxidative PPP. **a–f** HPD knockdown and control cells harboring an empty vector were tested for oxidative PPP flux (**a**), RNA biosynthesis (**b**) and NADPH/NADP^+^ ratio (**c**), glycolysis ratio (**d**), lactate production (**e**), as well as ROS level (**f**). The error bars represent mean values ± SD from three independent experiments (*0.01 < *p* < 0.05; **0.001 < *p* < 0.01; ***p < 0.001)

It has become clear that the PPP plays a critical role in regulating cancer cell growth by supplying cells with not only ribose-5-phosphate (R5P) but also nicotinamide-adenine dinucleotide phosphate (NADPH). R5P is the building block for nucleotide synthesis, while NADPH not only fuels macromolecular biosynthesis such as lipogenesis, but also functions as a crucial antioxidant to quench the ROS produced during rapid proliferation of cancer cells. However, the detailed signaling mechanisms by which cancer cells coordinate catabolic metabolism (tyrosine metabolism) and anabolic biosynthesis (nucleotide synthesis) to promote cancer cell proliferation and tumor growth remain largely unclear. With the cell systems described above, we found that knockdown of HPD resulted in reduced oxidative PPP flux (Fig. 2a), RNA biosynthesis (Fig. 2b), DNA synthesis (Fig. S2A, B), and NADPH/NADP^+^ ratio (Fig. 2c). We also observed increased glycolytic rate (Fig. 2d), lactate production (Fig. 2e), ROS levels (Fig. 2f), and intracellular ATP levels (Fig. S2C). Overexpression of HPD enhanced the NADPH/NADP^+^ ratio (Fig. S2D) and DNA synthesis (Fig. S2E), but decreased lactate production (Fig. S2F). These data together suggest that HPD rewires cell metabolism, especially PPP flux.

### HPD promotes PPP flux and tumor growth through upregulation of G6PD

**Fig. 3:**
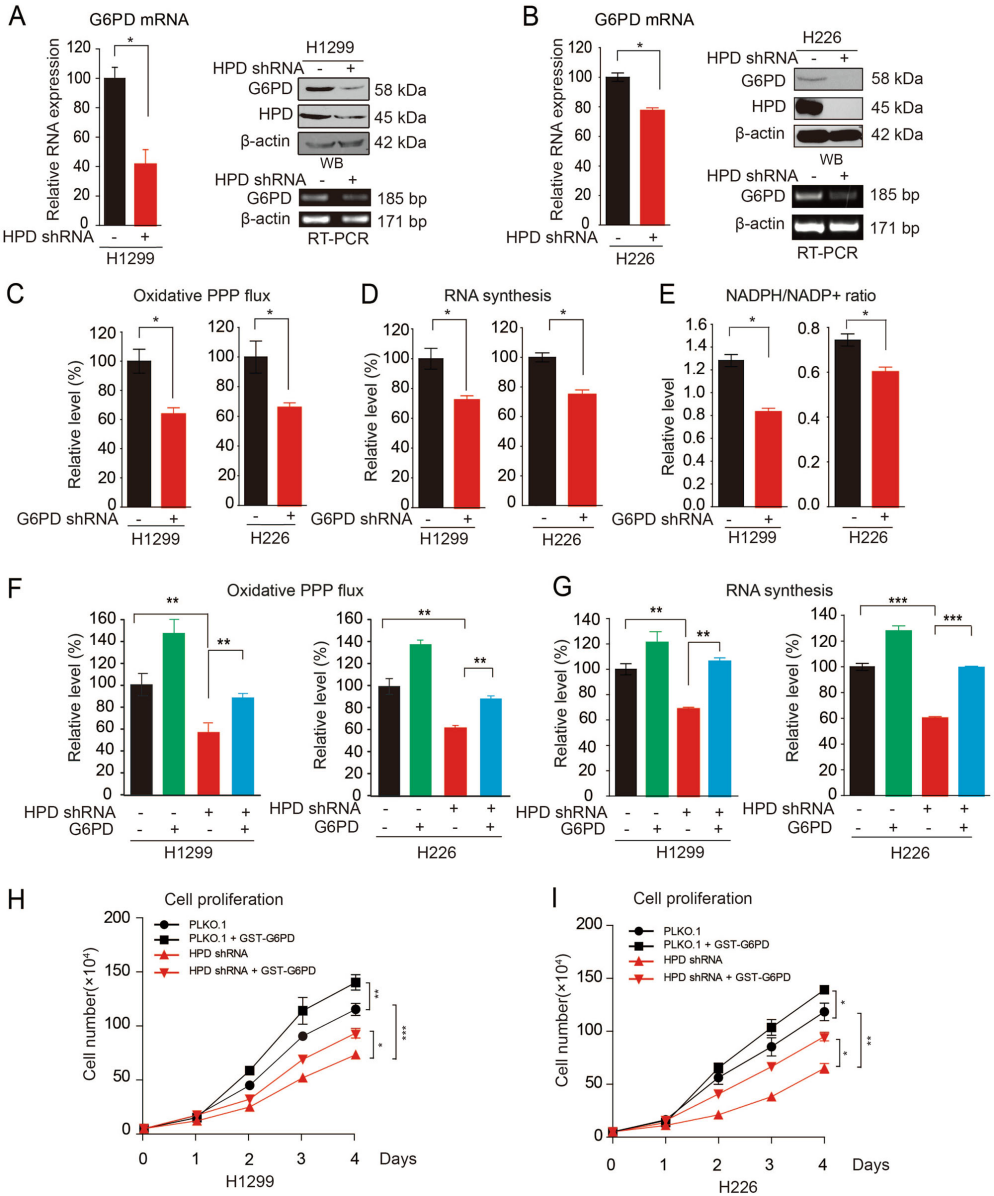
HPD contributes to cell proliferation through upregulation of G6PD. **a** Determination of G6PD mRNA expression levels by qRT-PCR (left), protein levels by western blotting (right upper), and mRNA expression levels by RT-PCR (right lower) in HPD-knockdown and control H1299 cells. **b** Determination of G6PD mRNA expression levels by qRT-PCR (left), protein levels by western blotting (right upper), and mRNA expression levels by RT-PCR (right lower) in HPD-knockdown and control H226 cells. **c**–**e** G6PD knockdown and control cells harboring an empty vector were tested for oxidative PPP flux (**c**), RNA biosynthesis (**d**), and NADPH/NADP^+^ ratio (**e**). **f**–**g** Overexpression of G6PD in HPD-knockdown cells restores decreased oxidative PPP flux (**f**) and RNA biosynthesis (**g**). **h**–**i** Cell proliferation rates determined by cell counting in human lung cancer H1299 (**h**) and H226 (**i**) cells with stable-knockdown HPD and overexpression of G6PD. The error bars represent mean values ± SD from three independent experiments (*0.01 < *p* < 0.05; **0.001 < *p* < 0.01; ***p < 0.001)

To address the role of HPD in promoting tumor growth mediated by PPP flux, we first compared the expression and activities of enzymes involved in PPP between systems with different HPD expression. Our results showed that G6PD mRNA and protein expression levels were decreased in HPD-knockdown cells (Fig. 3a, b). However, the 6PGD mRNA and protein expression levels were not affected (Fig. S3A). Moreover, exogenous expression of HPD significantly increased the G6PD enzyme activity, mRNA level and protein level in H1299, H226 lung cancer cells and 293T cells (Fig. S3B, D).

We next asked whether 6PGD is downstream of HPD in regulating PPP flux and cell proliferation. We first generated G6PD stable-knockdown H1299 cells (Fig. S3E), and found that G6PD knockdown suppressed cell proliferation (Fig. S3F), which was similar to the effect of knocking down HPD (Fig. 1e). Further examination showed that knocking down G6PD in H1299 and H226 cells resulted in reduced oxidative PPP flux (Fig. 3c), RNA biosynthesis (Fig. 3d), and NADPH/NADP^+^ ratio (Fig. 3e), as well as increased glycolytic rate (Fig. S3G), elevated lactate production (Fig. S3H), and ROS levels (Fig. S3I). These results demonstrate that G6PD has the same effect as HPD in reprogramming cell metabolism. In HPD-knockdown cell systems, we found that overexpression of G6PD restored the decreased oxidative PPP flux (Fig. 3f), RNA synthesis (Fig. 3g), increased lactate production (Fig. S3K), and increased ROS levels (Fig. S3L). Last, exogenous expression of 6PGD in HPD-knockdown cells rescued the decreased cell proliferation in both H1299 and H226 cells (Fig. 3h, i). Taken together, these results indicate that HPD promotes oxidative PPP flux through regulating G6PD expression.

### HPD promotes G6PD gene expression by controlling histone acetylation

**Fig. 4:**
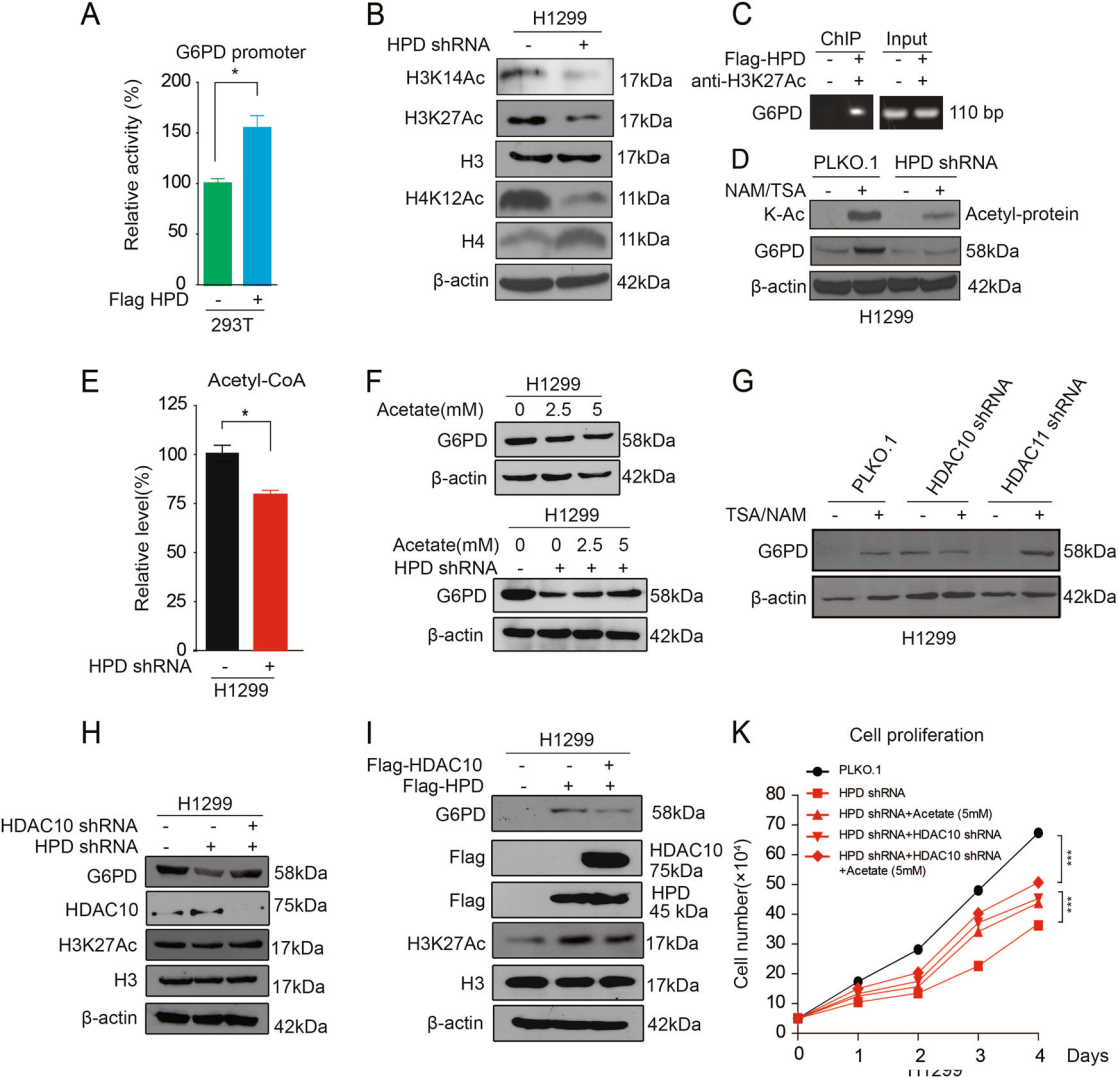
HPD promotes G6PD gene expression by controlling histone acetylation. **a** 293T cells were cotransfected with Renilla luciferase plasmid and a firefly luciferase reporter plasmid containing G6PD 3′UTR (indicated as pGL3-G6PD-3′UTR) with either control or Flag-HPD. Luciferase activity was conducted at 24 h after transfection. **b** H3 and H4 acetylation levels were analyzed in HPD-knockdown cells by western blotting. **c** The interaction between H3K27Ac and the promoter region of G6PD was examined by ChIP assay. **d** G6PD protein levels were analyzed by western blotting in HPD-knockdown H1299 cells and control vector cells with TSA/NAM treatment. **e** Acetyl-CoA levels were determined in HPD-knockdown H1299 cells. **f** H1299 cells treated with increasing concentrations of acetate were assayed for G6PD, and HPD-knockdown H1299 cells treated with increasing concentrations of acetate were assayed for G6PD. **g** G6PD protein levels were analyzed by western blotting in HDAC10 and HDAC11-Knockdown H1299 cells and control vector cells with TSA/NAM treatment. **h** G6PD protein levels and H3 acetyaltion level were analyzed by western blotting in HPD knockdown H1299 cells and control vector cells infected with lentivirus harboring HDAC10 shRNA. **i** G6PD protein levels and H3 acetylation levels were analyzed by western blotting in HPD stable-expressing H1299 cells and control vector cells that were transiently transfected with vectors encoding HDAC10. **j** Cell proliferation rates were determined by cell counting in HPD-knockdown H1299 cells treated with acetate or HPD-knockdown cells infected with lentivirus harboring HDAC10 shRNA. The error bars represent mean values ± SD from three independent experiments (*0.01 < *p* < 0.05; **0.001 < *p* < 0.01; ***p < 0.001)

To explore the mechanism of HPD on regulation of G6PD expression, we first found that exogenous expression of HPD would increase G6PD promoter activity (Fig. 4a). The role of histone acetylation and its involvement in the regulation of transcription has long been a topic of research in cell and molecular biology labs. Therefore, we hypothesized that HPD might regulate G6PD expression through histone acetylation levels. Indeed, we found that HPD knocking down decreased acetylation levels of H3K14, H3K27, and H4K12 (Fig. 4b). ChIP analysis indicated that HPD increases the H3K27Ac level on the promoter of the G6PD gene (Fig. 4c). Furthermore, we treated H1299 lung cancer cells with deacetylase inhibitors, nicotinamide (NAM) and trichostatin A (TSA). The data showed that the treatment induced expression of G6PD, while and knocking down of HPD weaken the upregulation of G6PD by the inhibitors (Fig. 4d). Together, these results suggest that HPD increases G6PD promoter activity via histone acetylation modification.

Histone acetylation occurs by the enzymatic addition of an acetyl group (COCH3) from acetyl coenzyme A (acetyl-CoA), which is catalyzed by histone acetyl transferases (HATs), whereas the reverse reaction is performed by histone deacetylases (HDACs). Interestingly, we observed that acetyl-CoA levels were decreased in HPD-knockdown cells compared with control cells (Fig. 4d). To characterize the role of epigenetic regulation by histone acetylation, we treated HPD-knockdown cells and control vector cells with different concentrations of acetate and found that exogenous acetate supplement rapidly increased G6PD expression in a dose-dependent manner in HPD knockdown cells, which is not the case in control vector cells. In addition, acetate supplement could not totally rescue the decreased G6PD level, even with amounts of acetyl-CoA exceeding physiological levels (Fig. 4f), which indicates that HATs or HDACs may be involved. Therefore, we next test whether HATs or HDACs are also responsible for the G6PD expression in HPD-knockdown cells. To do this, we constructed two novel “targeted” lentiviral shRNA libraries based on the whole human genome library from Open Biosystems, which target 50 out of 71 acetyltransferases and 36 out of 40 deacetylases, respectively. With these two libraries, we performed a G6PD expression screening assay using cell lysates from H1299 cells, with knockdown of particular acetyltransferases or deacetylases untreated or treated with NAM plus TSA. The screening data showed that HDAC10 acts as a potential deacetylase for regulation of G6PD expression (Fig. 4g). We next employed a luciferase reporter assay, demonstrating that HDAC10 can direct target G6PD promoter (Fig. S4B). To validate the role of HDAC10 targeting G6PD, we first generated HDAC10-knockdown cells and found increased levels of H3K27 acetylation, G6PD mRNA, and G6PD protein, as well as G6PD enzyme activity induced by HDAC10 deficiency (Fig. S4C–E). Furthermore, HDAC10 knockdown could rescue the decreased G6PD expression in HPD-knockdown cells (Fig. 4h). Similarly, overexpression of HDAC10 could block the increased G6PD expression in HPD-overexpression cells (Fig. 4i). In addition, although treatment with acetate did not affect H1299 cell proliferation (data not shown) and HDAC10 knockdown suppressed H1299 cell proliferation (Fig. S4F), acetate or HDAC10 shRNA treatments could significantly rescue the reduced proliferation of cells induced by HPD knockdown (Fig. 4k). Combined treatment with acetate and HDAC10 shRNA further restored the cell proliferation (Fig. 4k). Taken together, these results suggest that HPD regulates G6PD expression via histone acetylation modification.

### HDAC10 phosphorylation regulates G6PD expression

**Fig. 5:**
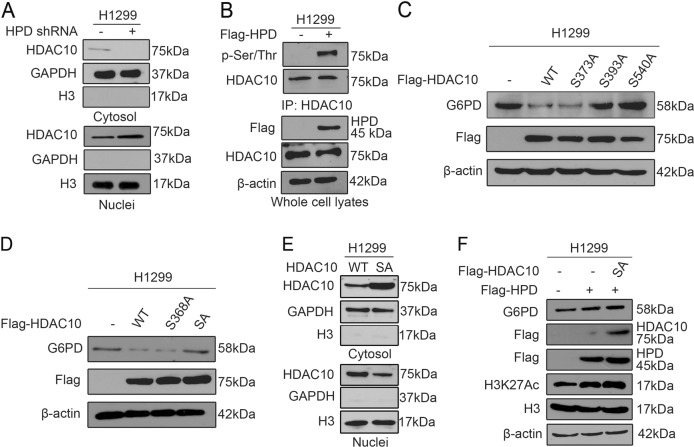
HPD promotes G6PD gene expression by controlling HDAC10 phosphorylation. **a** The purity of the cytosolic and nuclear fractions from knockdown and control cells harboring an empty vector was tested for HDAC10 localization. **b** Immunoprecipitation of HDAC10 and western blot to detect phosphorylation levels of HDAC10 in HPD stable-expressing H1299 cells and control vector cells. **c** G6PD protein levels were analyzed by western blotting in H1299 cells, which were transiently transfected with vectors encoding HDAC10 WT, S373, S393, and S540 mutants. **d** G6PD protein levels were analyzed by western blotting in H1299 cells, which were transiently transfected with vectors encoding HDAC10wt, S368, and double S393/540 mutants (SA). **e** G6PD protein and H3 acetyaltion levels were analyzed by western blotting in HPD stable-expressing H1299 cells that were transiently transfected with vectors encoding HDAC10 double S393/540 mutants (SA). **f** Purity of the cytosolic and nuclear fractions from H1299 cells, which were transiently transfected with vectors encoding HDAC10 WT and HDAC10 double S393/540 (SA) mutant

Next, we explored the mechanism underlying how HPD regulates G6PD via HDAC10. With the HPD-knockdown cell system, we observed HDAC10 nuclear localization in HPD-knockdown cells compared with control cells, where HDAC10 localized to the cytosol (Fig. 5a). Overexpression of HPD promoted HDAC10 cytoplasmic localization (Fig. S5A). Therefore, we hypothesized that HPD might regulate G6PD expression through regulating HDAC10 translocation. To explore the mechanism of HDAC10 translocation, we found that HPD promotes HDAC10 phosphorylation (Fig. 5b, S5B). Moreover, proteomics-based studies performed by Cell Signaling Technology revealed that HDAC10 can be phosphorylated at a group of phosphorylation residues (https://www.phosphosite.org/). We then performed mutational analysis and generated diverse serine-deficient mutants (S–A) to examine the effect of phosphorylation on HDAC10 localization. The substitution of S393 or S540 alone or together abolished the downregulation of G6PD by HDAC10 (Fig. 5c, d). Furthermore, our results showed that HDAC10 S393A, S540A, and double S393/540A (SA) mutants blocked HDAC10 nuclear translocation (Fig. 5e). To further confirm that the serine phosphorylation status on HDAC10 tightly correlated with the regulation of G6PD by HPD, we transfected HDAC10 mutants into HPD stable-expressing cells. Western blotting results showed that HDAC10 double S393/540A (SA) mutants fail to reduce the increased G6PD expression and H3K27 acetylation level (Fig. 5f, S5C). A luciferase reporter gene assay indicated that HDAC10 S393A, S540A, and double S393/540A (SA) mutants could not affect G6PD promoter activity (Fig. S4B). Taken together, these results suggest that HPD might regulate G6PD expression through controlling HDAC10 translocation, depending on its phosphorylation status.

### HPD regulates HDAC10 phosphorylation through AMPK activity mediated by LKB1

**Fig. 6:**
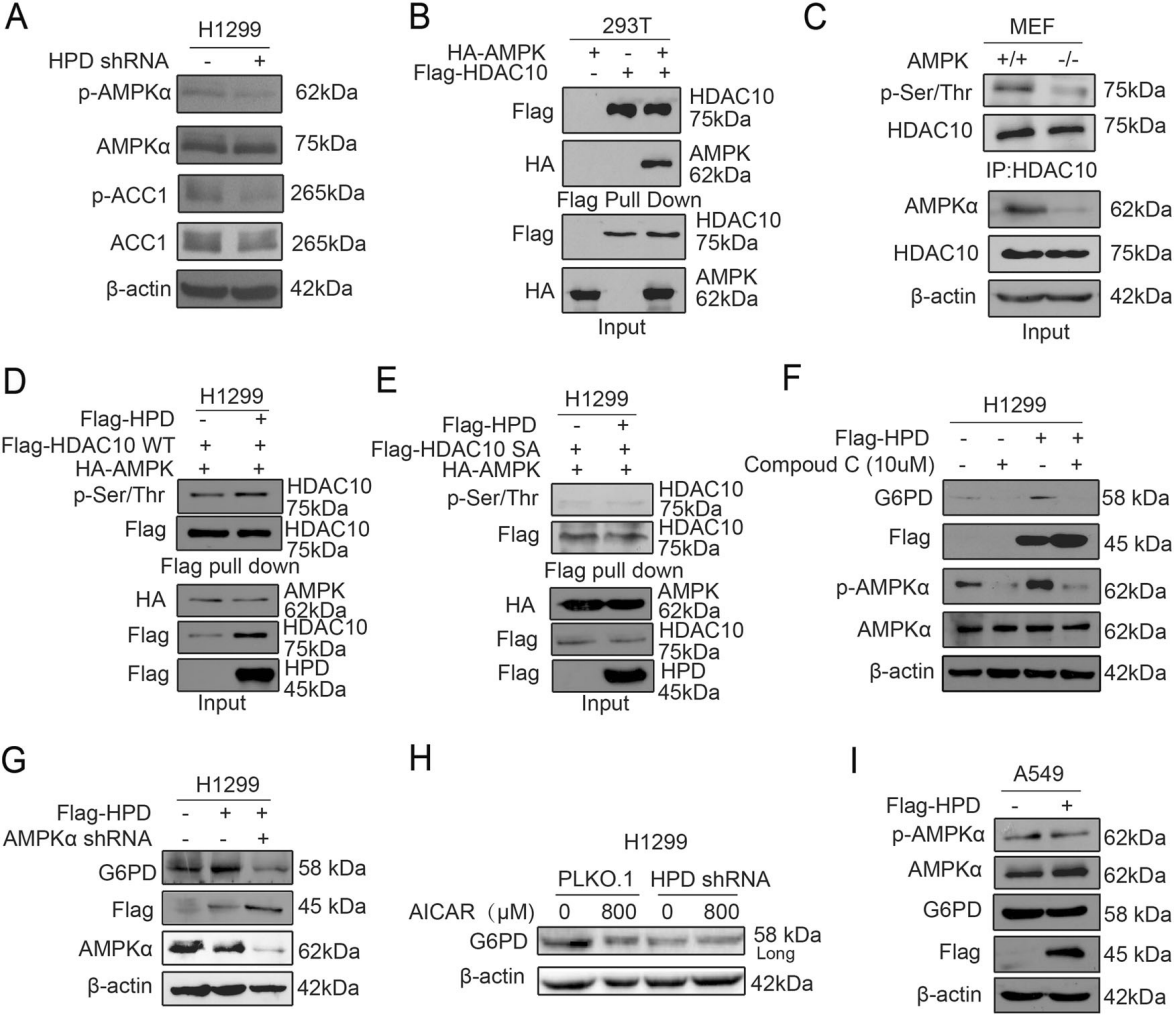
HPD promotes G6PD gene expression by controlling AMPK activity. **a** Cell lysates from HPD-knockdown H1299 cells were tested for phosphorylation levels of AMPK (pT172) and ACC1 (p79) by western blotting. **b** HEK293 cells were cotransfected with Flag-HDAC10 with or without HA-AMPK. HDAC10 was pulled down with Flag beads and coprecipitated proteins were analyzed by immunoblotting with anti-Flag or anti-HA antibody. The expression levels of transfected constructs were analyzed by immunoblotting. **c** Immunoprecipitation was conducted in AMPK (−/−) and AMPK (+/+) cell line MEF. In all, 2 mg of cell lysate was immunoprecipitated with anti-HDAC10 and immunoblotted using anti-HDAC10 or anti-phospho-(Ser/Thr). Sixty micrograms of cell lysate was used as an input control. **d** H1299 cells were cotransfected with Flag-HDAC10 WT with HA-AMPK in the presence of Flag-HPD. Flag-HDAC10 was pulled down with Flag beads and co-immunoprecipitated proteins were analyzed by immunoblotting with anti-Flag or anti-phospho-(Ser/Thr) antibody. The expression levels of transfected constructs were analyzed by immunoblotting. **e** H1299 cells were cotransfected with Flag-HDAC10 double S393/540 mutants (SA) with HA-AMPK in the presence of Flag-HPD. HDAC10 was pulled down with Flag beads and coprecipitated proteins were analyzed by immunoblotting with anti-Flag or anti-phospho-(Ser/Thr) antibody. The expression levels of transfected constructs were analyzed by immunoblotting. **f** G6PD protein levels were analyzed by western blotting in HPD stable-expressing H1299 cells and control vector cells with Compound C treatment. **g** G6PD protein levels were analyzed by western blotting in HPD stable-expressing H1299 cells infected with lentivirus harboring AMPK shRNA. **h** G6PD protein levels were analyzed by western blotting in HPD-knockdown H1299 cells and control vector cells with AICAR treatment. **i** Cell lysates from HPD-knockdown H1299 cells were tested for phosphorylation levels of AMPK (pT172), ACC1 (p79), and G6PD by western blotting

Finally, we explored the mechanism underlying how HPD regulates HDAC10 phosphorylation. We found that knockdown of HPD resulted in decreased AMPK activity and subsequently decreased inhibitory phosphorylation of acetyl-CoA carboxylase 1 (ACC1) (Fig. 6a, S6A). Furthermore, overexpression of HPD increased AMPK activity (Fig. S6B, C). We next examined whether AMPK signaling plays a role in regulation of HDAC10 by HPD. With lysates from cells transfected with Flag-HDAC10 and HA-AMPK2, pull-down assays showed that HDAC10 associated with AMPK2 (Fig. 6b, S6D). We also found that the HDAC10 phosphorylation levels were decreased in AMPK-knockout (KO) mouse embryonic fibroblast (MEF feeder cells) cells (Fig. 6c). Furthermore, in the presence of AMPK, overexpression of HPD enhanced HDAC10 wild-type (WT) phosphorylation, but not S393A, S540A, and double S393/540A (SA) mutants (Fig. 6d, e, S6E, F), Moreover, we found that inhibition of AMPK by Compound C or shRNA also led to decreased G6PD expression in HPD overexpression cells (Fig. 6f, g). Consistent results were obtained in cells with HPD knockdown in the presence of AMPK activation induced by 5-aminoimidazole-4-carboxamide ribonucleotide (AICAR) (Fig. 6h). Together, these data suggest that HPD regulates HDAC10 phosphorylation through suppression of AMPK activity.

Since LKB1 directly phosphorylates and activates AMPK^19^, we hypothesized that LKB1 may also be involved in the HPD cascade functions revealed above. We first examined the dependency of LKB1 in the regulation of AMPK by HPD. Indeed, data showed that, compared with data from LKB1 wild-type cells (Fig. 6a), knockdown or overexpression of HPD did not affect AMPK activity and G6PD expression in LKB1-deficient A549 and H157 cells (Fig. 6i, S6G, H). These results support our hypothesis that HPD regulates HDAC10 phosphorylation in the LKB1–AMPK axis, leading to regulating G6PD expression. Together, these results demonstrated that HPD regulates HDAC10 phosphorylation via the LKB1–AMPK axis, leading to upregulating G6PD expression.

## Discussion

Although HPD has been considered an important disease gene, whose deficiency and single mutation in the N-terminal region produces Type III tyrosinemia leading to hawkinsinuria^18,20^, its contribution to cell metabolism and tumor formation is not defined. The main finding of this study is that HPD acts as an activator of the PPP, which is vital for growth of lung cancer cells. The results reported here may not only help to understand the function and oncogenic potential of HPD, but also have implications in developing new strategies for cancer prevention and treatment.

We demonstrated that HPD is commonly upregulated in lung cancer and is important for cell proliferation and tumor growth through regulation of cell metabolism. This previous unappreciated function of HPD is independent of its metabolic tyrosine activity, but is achieved through promotion of PPP flux by increasing the expression of key enzyme G6PD. We propose a novel relationship between tyrosine metabolism and anabolic biosynthesis mediated by HPD–G6PD–PPP flux axis to coordinate tumor growth.

Tyrosine is metabolized by four enzymes to become fumarate and acetoacetate. Fumarate is an intermediate in the tricarboxylic acid cycle in which energy (ATP, adenosine triphosphate) is produced, and acetoacetate can be reduced to acetyl-CoA in ketolysis pathway. Acetyl-CoA is also dynamically associated with the acetylation modification to modulate protein functions^21,22^. Interestingly, we found that knockdown of HPD inhibits tyrosine catabolism, blocking the production of fumarate and acetoacetate, leading to the decrease of acetyl-CoA and histone acetylation, and finally suppression of G6PD expression. Moreover, we reveal that an exogenous acetate supplement rapidly increases G6PD expression in a dose-dependent manner in HPD knockdown cells, but it cannot totally rescue the decreased G6PD expression, indicating that an additional mechanism is involved. Since histone acetylation is also regulated by acetyltransferase(s) and deacetylase(s), we further screened out HDAC10 as responsible for the G6PD expression through regulating histone acetylation modification. These results are consistent with previous reports, which have shown that histone acetylation in mammalian cells is dependent on ATP–citrate lyase (ACL), an enzyme converting glucose-derived citrate into acetyl-CoA, regulating gene expression^23–25^.

To explore the physiological consequence of HPD on HDAC10, we monitored HDAC10 nuclear translocation in H1299 cells. Our finding shows that more HDAC10 protein was translocated into the nucleus in the absence of HPD. In addition, phosphorylation of HDAC10 affects its localization in the nucleus, which was confirmed by mutagenesis experiments. Many types of cellular stress can lead to AMPK activation. AMPK achieves its regulatory functions either via direct and rapid phosphorylation of the metabolic enzymes or induction of target genes^26–28^. AMPK is also a sensor of exercise and upstream kinase of class II HDACs that act as transcriptional repressors^29,30^. In this context, we found that HPD promotes AMPK activation-induced HDAC10 phosphorylation, leading to regulation of G6PD expression. Since LKB1 is a crucial upstream kinase of AMPK, LKB1–AMPK signaling plays a central role in regulation of cell metabolism, survival, and proliferation in response to nutrient and energy levels^31–34^. Indeed, we found that AMPK activity is not affected by HPD, nor was G6PD expression in LKB1-deficient lung cancer cells.

In summary, this study uncovers a new function of HPD in promoting PPP and tumor growth of lung cancer cells, and demonstrates that this previous unrecognized function of HPD is mediated through the G6PD–PPP flux axis. These findings provide a new mechanism accounting for the role of HPD in cancer progression and underscore the potential of targeting HPD as a novel strategy for cancer prevention and treatment.

## Methods

### Reagents and antibodies

Antibodies against Histone H3 (1:500 times dilution) (catalog number: ab1791), Histone H4 (1:500 times dilution) (catalog number: ab10158), Histone H3 (acetyl K27) (1:500 times dilution) (catalog number: ab4441), Histone H3 (acetyl K14) (1:500 times dilution) (catalog number: ab52946), Histone H4 (acetyl K12) (1:500 times dilution) (catalog number: ab46983), Goat Anti-Rabbit IgG H&L(HRP) (catalog number: ab6721), Phospho-serine/threonine (1:500 times dilution) (catalog number: ab17464), and HDAC10 (1:500 times dilution) (catalog number: ab108934) were from Abcam. Antibodies against AMPK (1:500 times dilution) (catalog number: 2532), Phospho-AMPK (T172) antibody(1:500 times dilution) (catalog number: 2535), Acetyl-CoA Carboxylase (1:1000 times dilution) (catalog number: 3676), Phospho-Acetyl-CoA Carboxylase (1:1000 times dilution) (catalog number: 3661), Acetylated-Lysine (1:1000 times dilution) (catalog number: 9681), HA-Tag (1:1000 times dilution) (catalog number: 3724), PGD (1:1000 times dilution) (catalog number: 13389), LaminB1 antibody (1:500 times dilution) (catalog numbe:13435), and β-actin (1:1000 times dilution) (catalog number: 3700) were from Cell Signaling. Glucose-6-Phosphate Dehydrogenase (1:1000 times dilution) (catalog number: 25413-1-AP), and Flag tag (1:1000 times dilution) (catalog number: 66008) were from Proteintech. An antibody against HPD (1:150 times dilution) (catalog number: GTX103994) was from GeneTex. Compound C (catalog number: HY-13418) were purchased from MedChem Express. 3X FLAG Peptide (catalog number: F4799) and ANTI-FLAG® M2 Affinity Gel (catalog number: A2220) were purchased from Sigma-Aldrich. Protein G Sepharose 4 Fast Flow (catalog number: 17061805) was purchased from GE. HPD, G6PD shRNA, and HAT and HDAC shRNA library were from Open Biosystems.

### Patient samples

The lung cancer tissue microarrays were purchased from Xi’an Aomei Biotechnology Co., Ltd. (Xi’an, China). These microarrays included 48 lung cancer tissues and 48 adjacent nontumor lung tissues. The relevant characteristics are shown in Table S2. The study protocol was approved by the Institute Research Ethics Committee at Nankai University.

### Evaluation of IHC staining

All specimens were examined by two investigators (Liu and Shan) who did not possess knowledge of the clinical data. In the case of discrepancies, a final score was established by reassessment on a double-headed microscope. Briefly, the IHC staining for 6PGD was semiquantitatively scored as “−” (negative, no, or <5% positive cells), “+” (5–50% positive cells), and “++” (more than 50% positive cells, considered as strongly positive). Only the cytoplasmic expression pattern was considered as positive staining.

### Cell lines

HEK293T cells were cultured in Dulbecco’s Modified Eagle Medium (DMEM) with 10% fetal bovine serum (FBS, ExCell Bio). H1299, H157, H1944, and H460 cells were cultured in RPMI 1640 medium with 10% FBS. All the cells were cultured at 37 °C and 5% CO_2_. Human lung adenocarcinoma epithelial cells A549 were cultured in DMEM with 10% FBS. Normal proliferating human bronchial epithelial cell line (BEAS-2B), a gift from Dr. Chenglai Xia (Guangzhou Medical University, Guangdong, China), was cultured in RPMI 1640 medium with 10% FBS. AMPK WT and KO MEF cells were gifts from Dr. Tongzheng Liu (Jinan University, Guangdong, China) and were cultured in DMEM with 10% FBS. The stable knockdown endogenous HPD, G6PD, HDAC10, and AMPK were achieved using lentiviral vectors harboring shRNA as previous described^35^.

### Western blot analysis

Cells were lysed with lysis buffer (1.5 M NaCl, 1 M HEPES[pH = 7.0], 1% NP40, 0.1 M Na_4_P_2_O_7_, 0.1 M NaF, 0.1 M Na_3_VO_4_, and protease inhibitor) on ice for 30 min and then centrifuged at 12,000 rpm for 15 min at 4 °C. Protein samples were separated by 12% SDS-PAGE and transferred onto PVDF membranes (Millipore). The membranes were blocked with 5% nonfat milk for 2 h and then incubated overnight at 4 °C with the primary antibody and 1 h at room temperature with a secondary antibody. Signals were detected using Luminol substrate solution.

### Real-time quantitative reverse transcription-PCR (qRT-PCR)

Total cellular RNA was extracted using the Eastep Super RNA Extract reagent Kit (Promega). cDNA was generated from purified RNA using PrimeScipt^TM^ RT reagent Kit (Takara) according to the manufacturer’s instructions. qRT-PCR was performed using SYBR Green (Biotool) on a Bio-rad CFX96 Realtime PCR System (Bio-rad). Relative HPD, G6PD, 6PGD, and HDAC10 mRNA (Table S3) levels were normalized to β-actin expression.

### Luciferase reporter gene assay

Luciferase reporter assay was performed using the Dual-Luciferase Reporter Assay System (Promega) according to the manufacturer’s instructions. When the cell density reaches 70% confluence, they were seeded in 24-well plates. For G6PD 3′UTR luciferase reporter gene assay, we cotransfected 293T cells with Flag-HPD or Flag-HDAC10 wt or Flag-HDAC10 mutants with pGL3-6PGD-3′UTR and Renilla luciferase plasmids using PEI (Sigma-Aldrich). Cells were lysed and assayed for luciferase activity 48 h after transfection. One hundered microliters of protein extracts were analyzed in a luminometer.

### Cell proliferation assay

Cell proliferation assays were performed by seeding 5 × 10^4^ cells in six-well plates. Cell growth was determined by cell numbers recorded at 0–4 days after being seeded.

### Tumor formation in nude mice

Nude mice (nu/nu, male 4–6-week-old) were subcutaneously injected with 20 × 10^6^ H1299 cells harboring an empty vector on the left flank, and cells with stable knockdown of endogenous HPD on the right flank; ten mice were included in each experiment, and were performed according to the institutional ethical guidelines for animal experiments. Nude mice (nu/nu, male 4–6-week-old) were employed for tumorigenicity analysis. The tumorigenicity of H1299 cells harboring empty vector and cells with stable knockdown of endogenous HPD cells was measured. Cells were harvested by trypsinization, washed twice with sterile PBS and resuspended at 100 × 10^6^ cells/ml. Then, 0.2-ml aliquots were injected subcutaneously into ten female nude mice, with 1299 cells harboring an empty vector on the left flank, and cells with stable knockdown of endogenous h6PGD on the right flank. Tumor growth was recorded by measurement of two perpendicular diameters, using the formula 4π/3 × (width/2)^2^ × (length/2). The tumors were harvested and weighed at the experimental endpoint, and the masses of tumors (g) derived from cells with and without stable knockdown of endogenous HPD. Statistical analyses have been done by comparison in relation to the control group with a two-tailed paired Student’s *t* test.

### Ethics approval and consent to participate

This study was carried out in accordance with the recommendations of requirements of the Ethical Review System of Biomedical Research Involving Human by National Health and Family Planning Commission of China, Nankai University and Jinan University Ethics Committee with written informed consent from all subjects. All subjects gave written informed consent in accordance with the Declaration of Helsinki. Approval of use of mice and designed experiments was given by the Laboratory Animal Ethics Committee Jinan University.

### Oxidative PPP flux assay

Oxidative PPP flux assay was used as ^14^CO_2_ release as described previously^35^. In brief, a 6-cm dish with cells was placed in a 10-cm dish with two sealed pinholes on the top. Cells on the 6-cm dish were treated with 2 mL of medium containing [1-^14^C]- or [6-^14^C]-glucose at 37 °C for 3 h, respectively. We then injected 0.3 mL of 50% TCA through one of the holes into cells to stop the PPP flux, and at the same time injected 0.3 mL of hyamine hydroxide into a small cup placed on the 10-cm dish through the second hole for trapping ^14^CO_2_ release. We sealed each dish with parafilm and placed the dish at room temperature for overnight. Hyamine hydroxide in the small cup was dissolved into 60% methanol and directly subjected to scintillation counting.

### RNA biosynthesis assay

In total, 8 × 10^5^ cells were seeded in a six-well plate, 24 h prior to treatment, and then incubated in complete medium with 2 µCi/mL of D-[U-^14^C]-glucose for 2 h. RNA was extracted using RNeasy columns (Qiagen). ^14^C-RNA was assayed by scintillation counter and normalized by the amount of total RNA.

### Microscopic analysis of EdU incorporation for DNA synthesis

The cells were incubated with EdU for 2 h. After fixation and permeabilization, the incorporated EdU was visualized by means of a click reaction using 1 × Apollo (Ribobio) staining reaction solution for 30 min at room temperature (RT). The nuclear DNA was stained with 1 × Hoechst33342 (30 min, RT). The images were obtained by ZEISS AXIOVERT A1 microscope. The data were analyzed using ZEN image analysis software.

### NADPH/NADP^+^ ratio

NADPH/NADP^+^ ratio were measured by a Colorimetric Assay Kit (Sigma-Aldrich) as described previously^35^. In brief, 2 × 10^6^ cells were trypsinized and washed with PBS, and lysed with 200 μL of NADP^+^ (or NADPH) extraction buffer. Lysed cells were incubated at 60° for 5 min, then added 20 μL of assay buffer, and 200 μL of the counter NADPH (or NADP^+^) extraction buffer was added to neutralize the extracts. The extracts were centrifuged at 12,000 rpm for 5 min, and the supernatants were used to check the NADPH/NADP^+^ ratio according to the manufacturer’s protocol. The absorbance at 565 nm from the reaction mixture was measured by plate reader at 0 and 30 min.

### Glycolytic rate

In total, 1 × 10^6^ cells were incubated in 1 mL of Krebs buffer without glucose for 30 min at 37 °C. The Krebs buffer was then replaced with Krebs buffer containing 10 mM glucose with 10 µCi of ^3^H-glucose. Following incubation for 1 h at 37 °C, 50 µL of medium were transferred to uncapped PCR tubes containing 50 µl of 2 N HCl. The PCR tube was transferred into a 1.5-mL tube. The 1.5-mL tube was sealed for diffusion for 24 h at 37 °C. The amounts of diffused ^3^H_2_O were determined by scintillation counting and indicated the glycolytic rate.

### Lactate production

Cellular lactate production was measured with a colorimetric-based lactate assay kit (MBL) In brief, we seeded cells in a six well-plate and incubated at 37 °C for overnight. Media on cells was replaced with phenol red-free RPMI medium without FBS when the cells were 50% confluent. The plate was then incubated for 1 h at 37 °C. After incubation, 1 mL of media from each well was assessed using the lactate assay kit. Cell numbers were counted by a microscope.

### Intracellular ATP assay

Intracellular ATP concentration was measured by an ATP Colorimetric/Fluorometric Assay Kit (Sigma-Aldrich) as previously described^35^. In brief, 1 × 10^6^ cells were trypsinized and resuspended in ultrapure water. Luminescence was measured by a spectrofluorometer (SpectraMax Gemini; Molecular Devices) immediately after the addition of ATP enzyme mix to the cell suspension.

### Intracellular ROS production

The amount of intracellular ROS was measured by detecting dichlorodihydrofluorescein, which is the cleavage product of carboxy-H_2_DCFDA (Invitrogen) by ROS. In total, 2 × 10^5^ cells were seeded in a six-well plate. Twenty-four hours after seeding, cells were washed with PBS and loaded with 5 μM carboxy-H_2_DCFDA for 30 min. The cells were harvested, resuspended in PBS, and analyzed using a FACS (BD Biosciences; excitation and emission at 490 and 530 nm, respectively).

### 6PGD and G6PD assays

6PGD and G6PD enzyme activity is determined by the NADPH production rate in assay buffer (0.1 mM NADP^+^, 1 mM MgCl_2_, and 50 mM Tris, pH 8.1) with 0.2 mM 6-phosphogluconate for 6PGD activity and in assay buffer with 0.2 mM glucose 6-phosphate, 0.2 mM 6-phosphogluconate for combined dehydrogenase activity of G6PD and 6PGD. Since a product of G6PD, 6-phosphogluconolactone, is rapidly hydrolyzed to a substrate of 6PGD, 6-phosphogluconate, in cells. The activity of G6PD is obtained by subtracting that of 6PGD from the combined dehydrogenase activity. In total, 10 μg of cell lysates or 1 μg of recombinant protein was added and the reaction was then initiated by adding NADP^+^. The increase in 340-nm absorbance (OD_340_) as a measure of NADPH production was obtained every 20 s for 10 min on a DU800 Spectrophotometer (Beckman Coulter).

### Construction of HDAC10 and G6PD 3′UTR vector

Genomic DNA was extracted from 293T cells using the TIANamp Genomic DNA kit (Tiangen) according to the manufacturer’s instructions. High-fidelity PCR (TAKARA) was used to amplify the G6PD promoter sequence from the genomic DNA as the following: (20 μL) were prepared, each containing 2.0 μL of 10 × PCR buffer, 200 μM each of dNTPs, 0.5 U Ex Taq, 20 ng of DNA, and 1.0 μM of G6PD promoter primer (Supplementary Table 4). The PCR thermal conditions were performed following the manufacturer’s protocols. The amplified products were inserted into the pMD-19T vector and verified by DNA sequencing. The pENTR-HDAC10 plasmid with C-terminal Flag tag was purchased from the Vigene Bioscience company, the HDAC10 single-point mutants (S368A, S373A, S393A, and S540A) were generated by site-direct mutagenesis (Fast Mutagenesis System, TransGen).

### Bioinformatics analysis

The public data sets GSE19804 and the human protein atlas (https://www.proteinatlas.org) dataset were used for bioinformatics analysis. Kaplan–Meier Plotter (http://kmplot.com/analysis/index.php?p=background) was used for overall survival.

### Statistical analysis

Statistical analyses were performed using Student’s *t* test. All data were obtained from three independent experiments performed in triplicate and were presented as the mean ± standard error. *P* < 0.05 was considered to indicate a statistically significant difference.

## Supplementary information

Supplemental Information

Supplemental Figure 1

Supplemental Figure 2

Supplemental Figure 3

Supplemental Figure 4

Supplemental Figure 5

Supplemental Figure 6

Supplementary Table 1

Supplementary Table 2

Supplementary Table 3

Supplementary Table 4
