# Correction: 4-hydroxyphenylpyruvate dioxygenase promotes lung cancer growth via pentose phosphate pathway (PPP) flux mediated by LKB1-AMPK/HDAC10/G6PD axis

**DOI:** 10.1038/s41419-025-07459-6

**Published:** 2025-02-26

**Authors:** Changliang Shan, Zhaoliang Lu, Zhen Li, Hao Sheng, Jun Fan, Qi Qi, Shuangping Liu, Shuai Zhang

**Affiliations:** 1 State Key Laboratory of Medicinal Chemical Biology, College of Pharmacy and Tianjin Key Laboratory of Molecular Drug Research, Nankai University, 300350 Tianjin, China; 2 The First Affiliated Hospital, Biomedical Translational Research Institute, Jinan University, 510632 Guangzhou, Guangdong China; 3 Department of Clinical Biological Resource Bank, Guangzhou Institute of Pediatrics, Guangzhou Women and Children’s Medical Center, Guangzhou Medical University, 510623 Guangzhou, Guangdong China; 4 Department of Medical Biochemistry and Molecular Biology, School of Medicine, Jinan University, 510632 Guangzhou, Guangdong China; 5 Department of Pharmacology, School of Medicine, Jinan University, 510632 Guangzhou, Guangdong China; 6 Department of Pathology, Medical School, Dalian University, 116622 Dalian, Liaoning China; 7 School of Integrative Medicine, Tianjin University of Traditional Chinese Medicine, 301617 Tianjin, China

Correction to: *Cell Death & Disease* 10.1038/s41419-019-1756-1, published online 8 July 2019

We found two errors in the graph of the article. First, in Supplemental Fig. 2A. The Overlap figure is wrongly used during the figure assembly process, that’s because we found the overlay is inconsistent with EdU and DAP’s picture. The overlap of shHPD of Replicaiton 1 was wrongly used the overlap of shHPD of Replicaiton 2 during the Supplemental Fig. 2a assembly process. Now, the error has now been corrected. However, I would like to mention that this error does not affect the conclusions of the study. Second, in Figure 5a. We carefully checked our original data record and found that the both blank band in Figure 5A were wrongly used during the figure assembly process. Now, the error has now been corrected and all raw data are provided. Please see the revised figures and and full-gel scans of western blots. However, I would like to mention that this error does not affect the conclusions of the study.

Originally published Supplemental Fig 2a and Fig. 5a
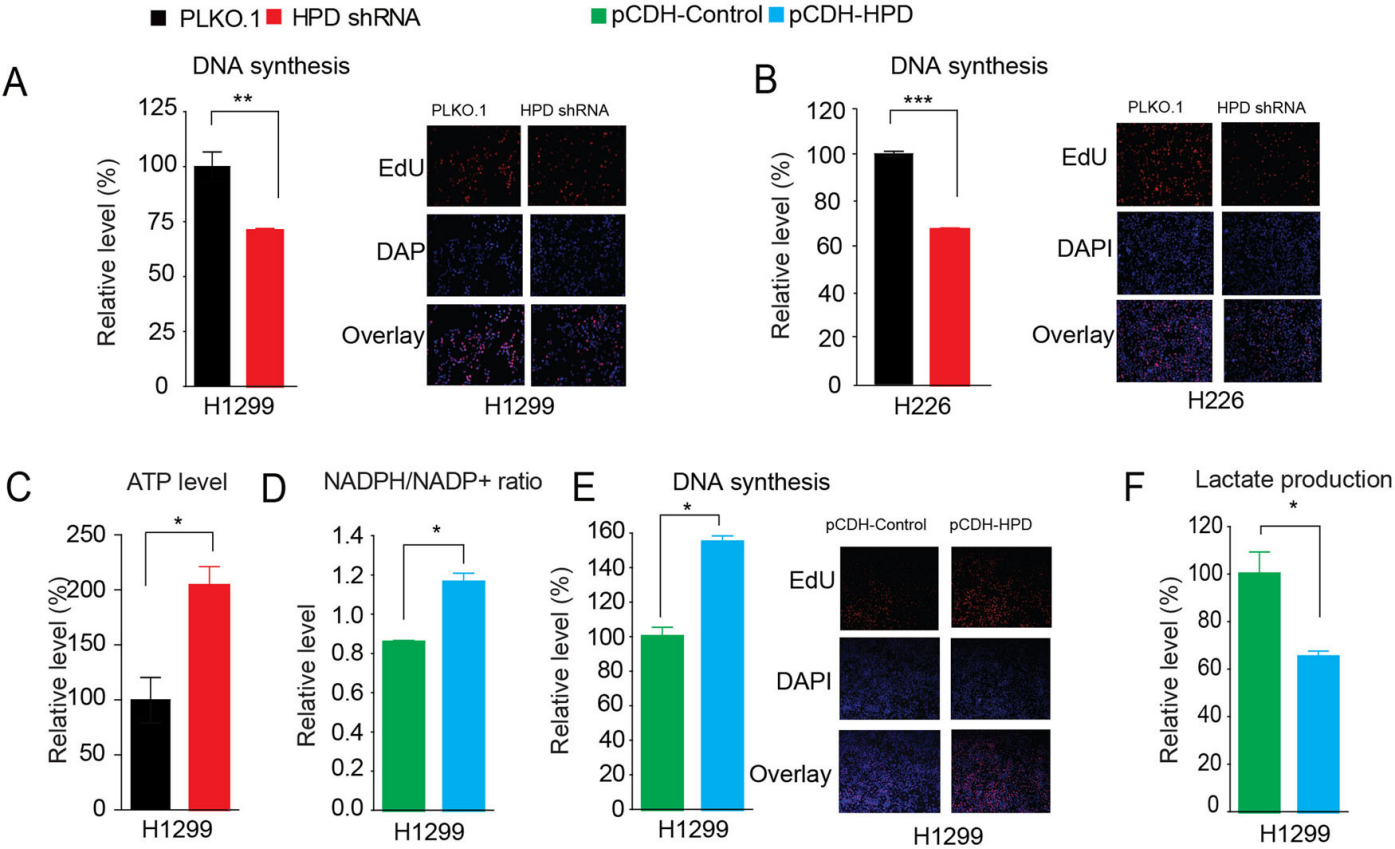

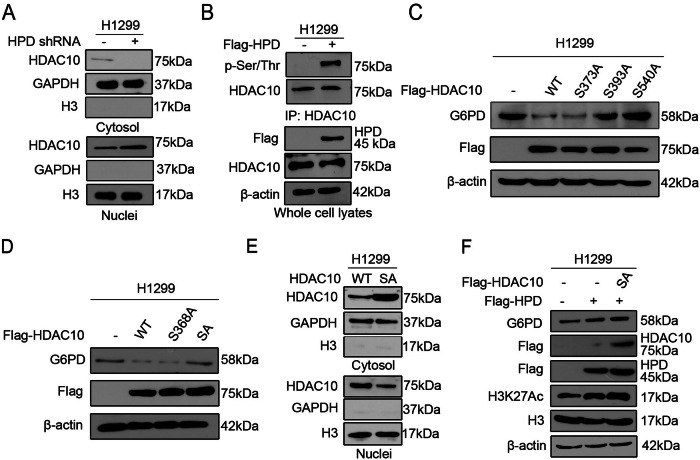


Corrected Supplemental Fig 2a and fig. 5a
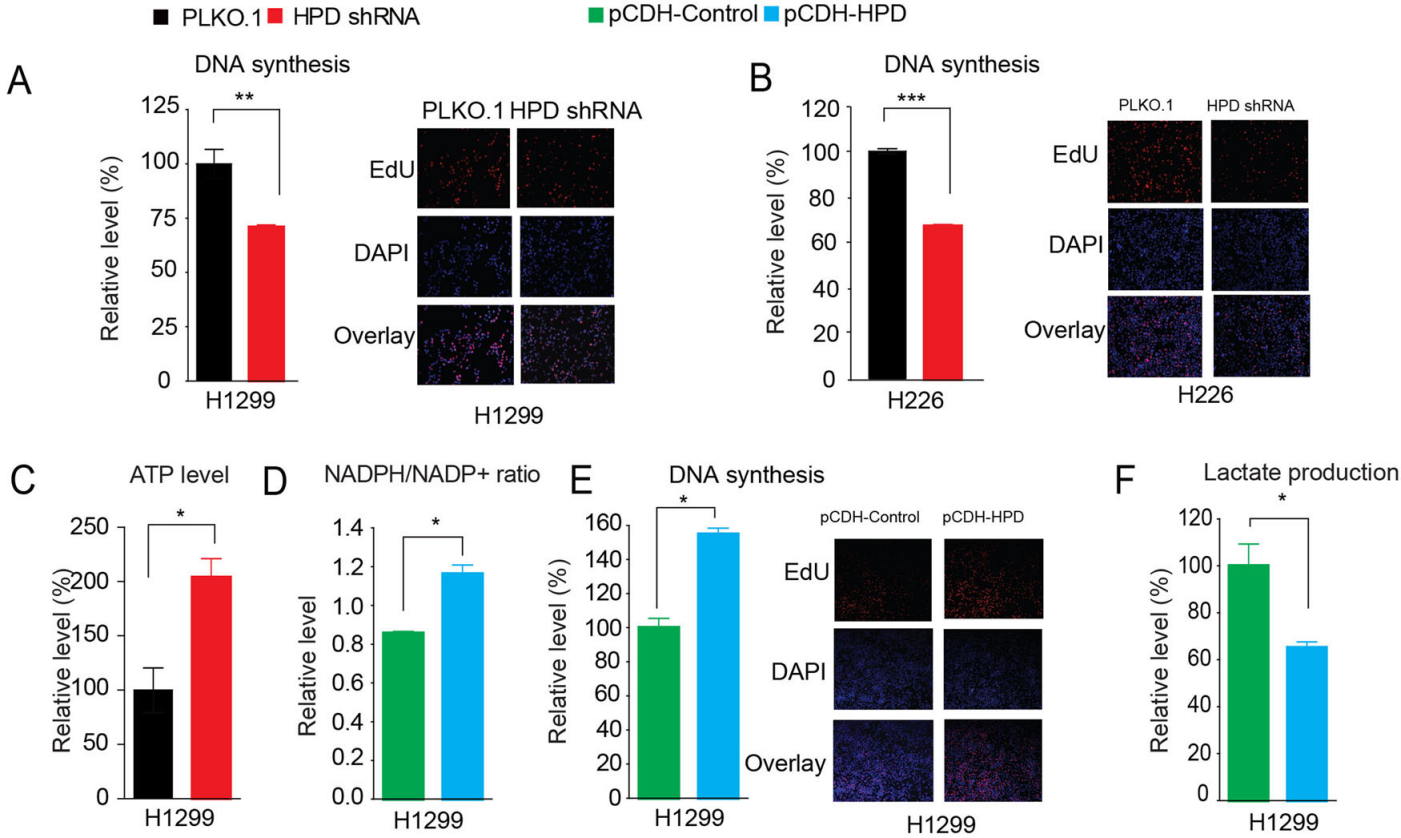

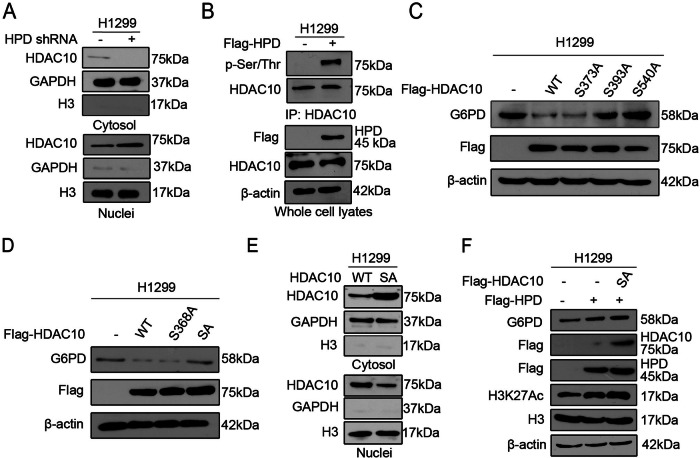


## Supplementary information

Original data V1

Supplemental Figure 2

