# Supplementary material for: 4-hydroxyphenylpyruvate dioxygenase promotes lung cancer growth via pentose phosphate pathway (PPP) flux mediated by LKB1-AMPK/HDAC10/G6PD axis
Source: Cell Death Dis. 2019 Jul 8;10(7):525. doi: 10.1038/s41419-019-1756-1 (PMC6614486; doi:10.1038/s41419-019-1756-1)
Supplement: Supplementary file 1 — Supplemental Information [file 41419_2019_1756_MOESM1_ESM.docx]

**Supplemental Information**

Supplemental Figures Legends

**Supplementary Figure 1 HPD expression is evaluated in lung cancer and is important for cancer cell proliferation and tumor growth. Related to Figure 1.**

(A) Kaplan–Meier curves of overall survival in lung cancer patients with high and low expression of HPD, calculated from (http://kmplot.com/analysis/). (B) Cell proliferation rates were determined by cell counting in HPD knock down BEAS-2B cells. (C) G6PD and HPD were determined by western blotting in HPD knock down BEAS-2B cells.

**Supplementary Figure 2 HPD reprograms oxidative PPP. Related to Figure 2.**

(A) Knock down HPD decreases DNA synthesis in H1299 cells. (B) Knock down HPD decreases DNA synthesis in H226 cells. (C) Knock down HPD increases ATP level in H1299 cells. (D) Overexpress HPD increases NADPH/NADP^+^ level in H1299 cells. (E) Overexpress HPD increases DNA synthesis in H1299 cells. (F) Overexpress HPD decreases lactate product in H1299 cells. The error bars represent mean values +/- SD from three independent experiments (*: 0.01<p<0.05; **: 0.001<p<0.01; ***: p<0.001).

**Supplementary Figure 3 HPD contributes to cell proliferation regulation through G6PD. Related to Figure 3.**

(A) Real-time PCR and Western blotting analysis of 6PGD RNA and protein expression in H1299 cells when knock down HPD. (B-C) Enzyme activity and Real-time PCR analysis of G6PD activity and RNA expression in H1299 cells when stable express HPD. (D) Western blotting analysis of G6PD protein expression in H1299, H226 and 293T cells when stable express HPD. (E) Western blotting was performed to determine the expression of G6PD protein in G6PD knock down H1299 cells. (F) Cell proliferation rates were determined by cell counting in G6PD knock down H1299 cells. (G-I) G6PD Knock down H1299 cells and control vector cells were tested for glycolysis ratio (G), lactate production (H), as well as ROS level (I). (K) Overexpress of 6PGD in HPD knock down cells would restore lactate product in H1299 and H226 cells. (L) Overexpress of 6PGD in HPD knock down cells would restore ROS level in H1299 and H226 cells. Error bars represent mean values±SD from three independent experiments (* *P<*0.05; ***P*<0.01; *** *P*<0.001).

**Supplementary Figure 4 HPD promotes G6PD gene expression by controlling Histone acetylation modification.** **Related to Figure** 4.

(A) Schematic representation of Tyrosine metabolism. (B) 293T cells were co-transfected with Renilla luciferase plasmid and a firefly luciferase reporter plasmid containing either G6PD 3`UTR (indicated as pGL3-G6PD-3`UTR) with either control or Flag-HDAC10 WT, S393, S540 and double S393/540 mutants. Luciferase activity was conducted 24h after transfection. (C) H3 acetylation and G6PD levels were analyzed in HDAC10 knock down cells by western blotting. (D) Real-time PCR analysis of G6PD RNA expression in HDAC10 knock down H1299 cells. (E) Enzyme activity assay analysis of G6PD activity in HDAC10 knock down H1299 cells. (F) Cell proliferation rates determined by cell counting in human lung cancer H1299 cells with stable knockdown of HPD. Error bars represent mean values±SD from three independent experiments (* *P<*0.05; ***P*<0.01; *** *P*<0.001).

**Supplementary Figure 5 HPD promotes G6PD gene expression by controlling HDAC10 phosphorylation. Related to Figure 5.**

**(A)** The purity of the cytosolic and nuclear fractions from HPD overexpree and control cells harboring an empty vector were tested for HDAC10 location. (B) Immunoprecipitation of HDAC10 and Western blot to detect phosphorylation levels of HDAC10 in HPD express 293T cells and control vector cells. (C) G6PD protein and H3 acetyaltion levels were analyzed by western blotting in HPD stable express H1299 cells were transiently transfected with vectors encoding HDAC10 S393 and S540 mutants. Error bars represent mean values±SD from three independent experiments (* *P<*0.05; ***P*<0.01; *** *P*<0.001).

**Supplementary Figure 6 HPD promotes G6PD gene expression by controlling AMPK activation. Related to Figure 6.**

**(A)** Cell lysates from HPD knock down H226 cells were tested for phosphorylation levels of AMPKα (pT172) and ACC1 (p79) by Western blotting. (B) Cell lysates from HPD stable express H1299 cells were tested for phosphorylation levels of AMPKα (pT172) and ACC1 (p79) by Western blotting. (C) Cell lysates were tested for phosphorylation levels of AMPKα (pT172) and ACC1 (p79) by Western blotting from HEK293 cells were transiently transfected HPD. (D) HEK293 cells were cotransfected with HA-AMPK with or without Flag-HDAC10. AMPK was pulled down with flag beads and coprecipitated proteins were analyzed by immunoblotting with anti-Flag or anti-HA antibody. The expression levels of transfected constructs were analyzed by immunoblotting. (E) H1299 cells were cotransfected with Flag-HDAC10 S393A with HA-AMPKα in the presence of Flag-HPD. Flag-HDAC10 S393A was pulled down with Flag beads and coprecipitated proteins were analyzed by immunoblotting with anti-Flag or anti-phospho-(Ser/Thr) antibody. The expression levels of transfected constructs were analyzed by immunoblotting. (F) H1299 cells were cotransfected with Flag-HDAC10 S540A with HA-AMPKα in the presence of Flag-HPD. Flag-HDAC10 S540A was pulled down with Flag beads and coprecipitated proteins were analyzed by immunoblotting with anti-Flag or anti-phospho-(Ser/Thr) antibody. The expression levels of transfected constructs were analyzed by immunoblotting. (G) Cell lysates from HPD knock down A549 cells were tested for phosphorylation levels of AMPKα (pT172) and ACC1 (p79) by Western blotting. (H) Cell lysates were tested for phosphorylation levels of AMPKα (pT172) and ACC1 (p79) by Western blotting from H157 cells were transiently transfected HPD. (I) Cell proliferation rates determined by cell counting in human lung cancer A549 cells with stable knockdown of HPD. The error bars represent mean values +/- SD from three independent experiments (*: 0.01<p<0.05; **: 0.001<p<0.01; ***: p<0.001).
