# Supplementary figures and images for: 4-hydroxyphenylpyruvate dioxygenase promotes lung cancer growth via pentose phosphate pathway (PPP) flux mediated by LKB1-AMPK/HDAC10/G6PD axis
Source: Cell Death Dis. 2019 Jul 8;10(7):525. doi: 10.1038/s41419-019-1756-1 (PMC6614486; doi:10.1038/s41419-019-1756-1)

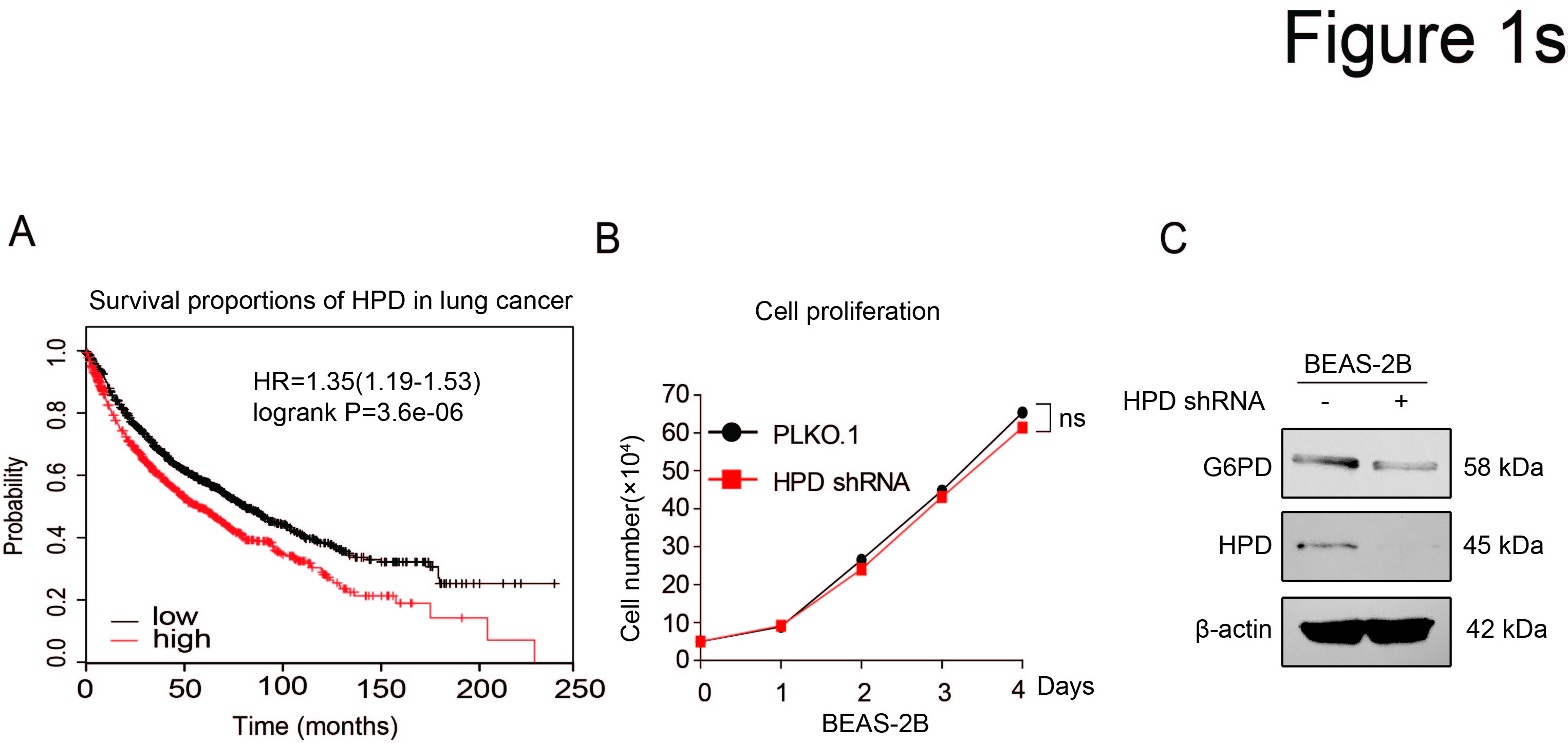

Supplement: Supplementary file 2 — Supplemental Figure 1 [file 41419_2019_1756_MOESM2_ESM.jpg]

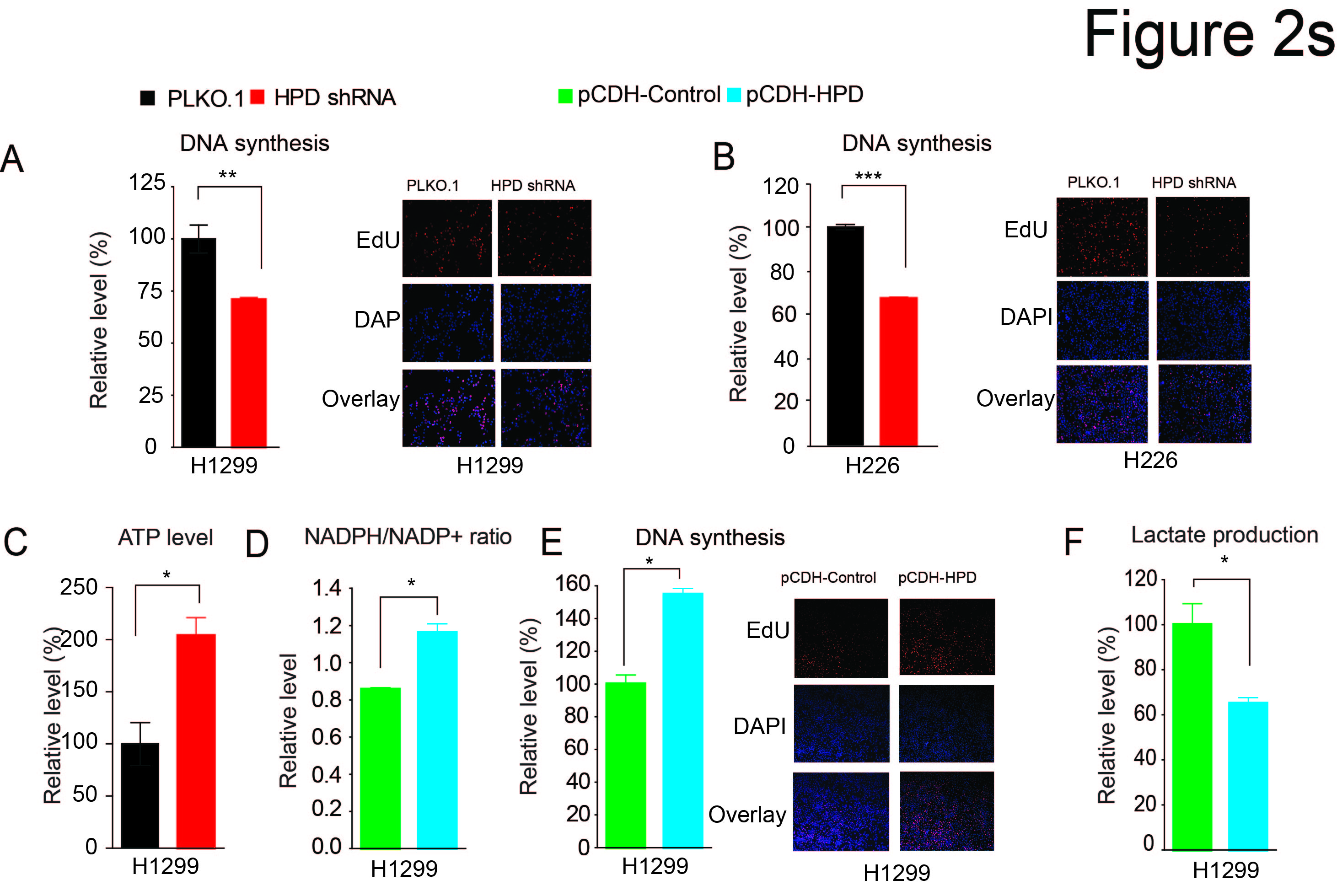

Supplement: Supplementary file 3 — Supplemental Figure 2 [file 41419_2019_1756_MOESM3_ESM.jpg]

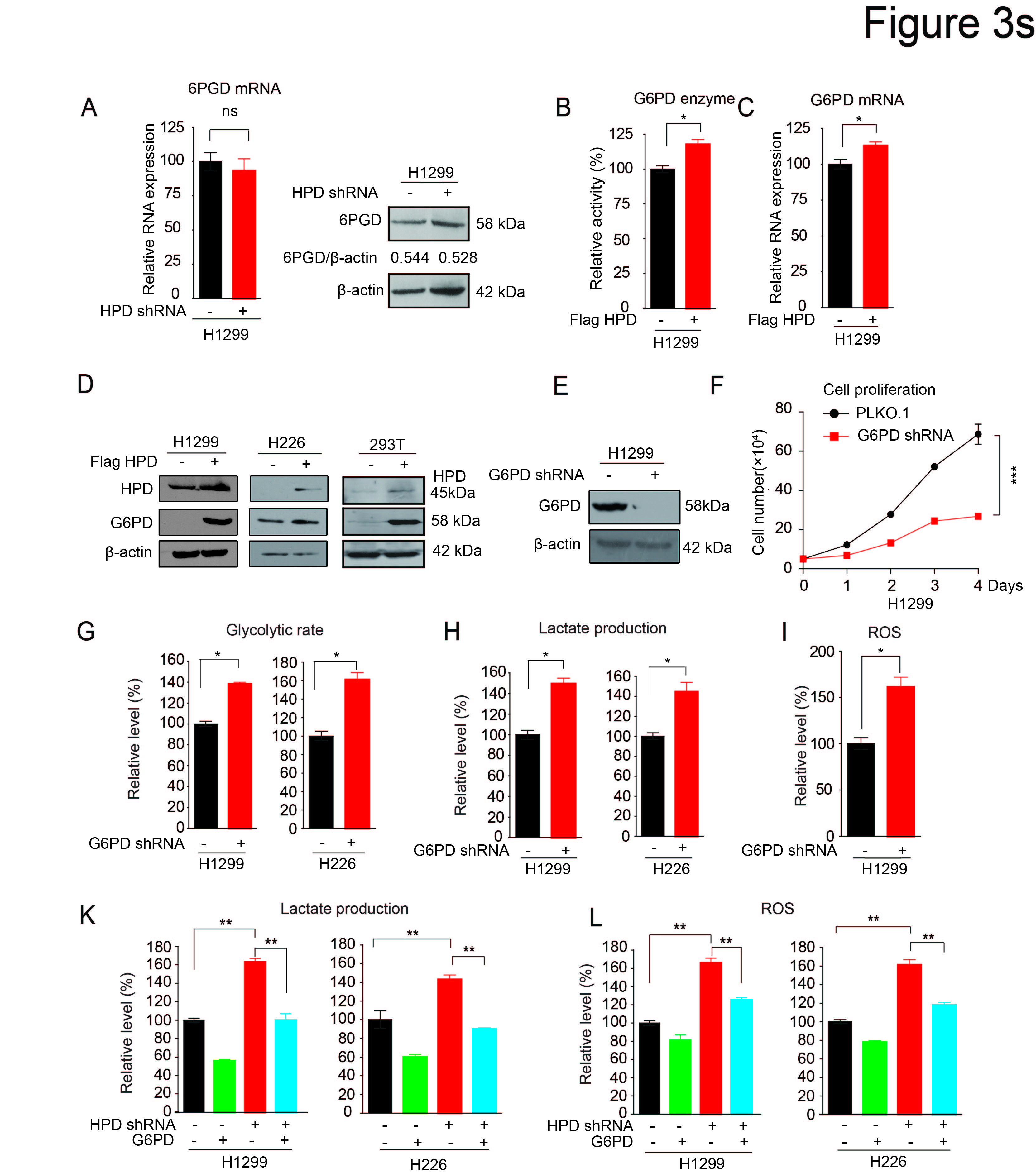

Supplement: Supplementary file 4 — Supplemental Figure 3 [file 41419_2019_1756_MOESM4_ESM.jpg]

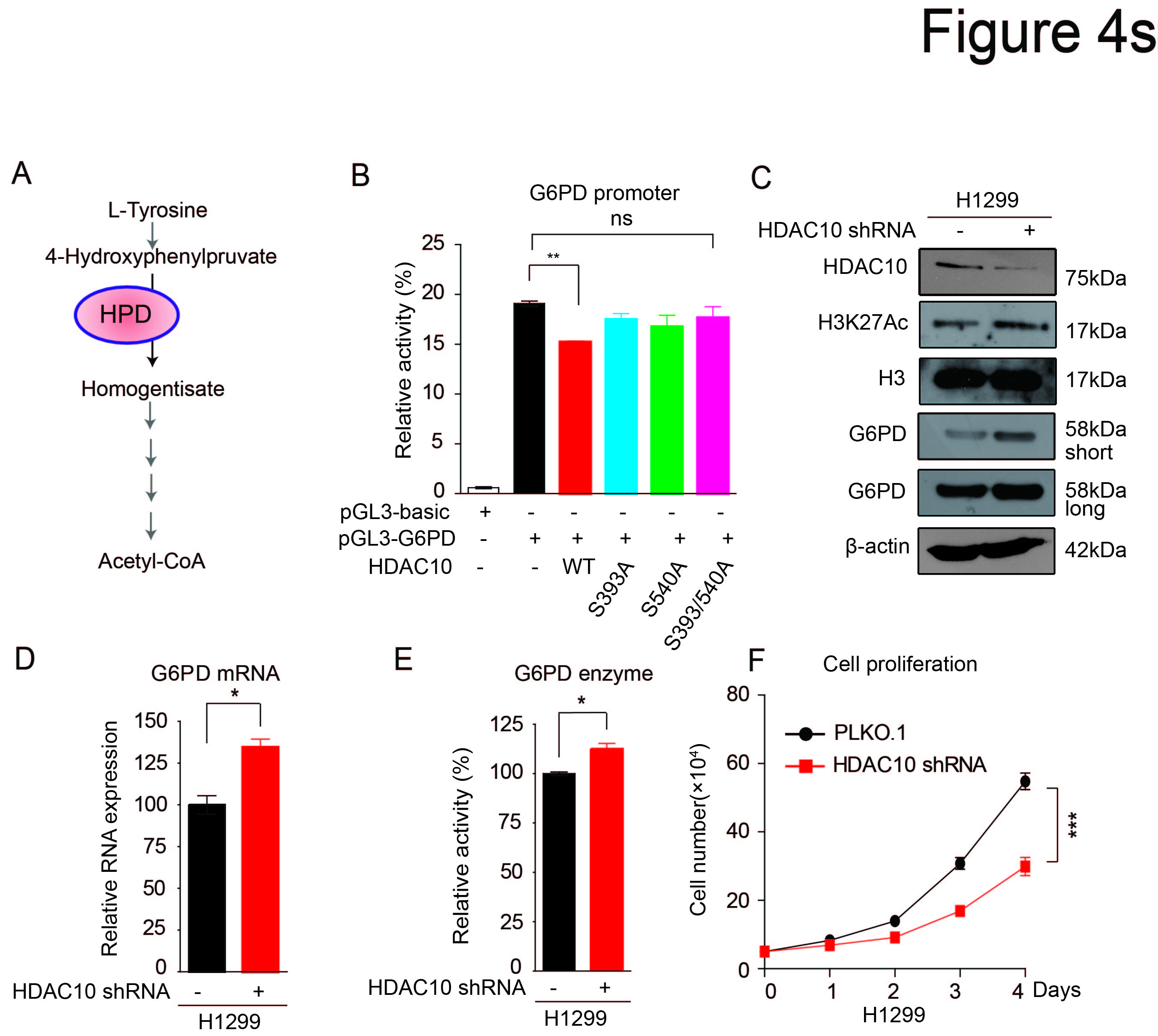

Supplement: Supplementary file 5 — Supplemental Figure 4 [file 41419_2019_1756_MOESM5_ESM.jpg]

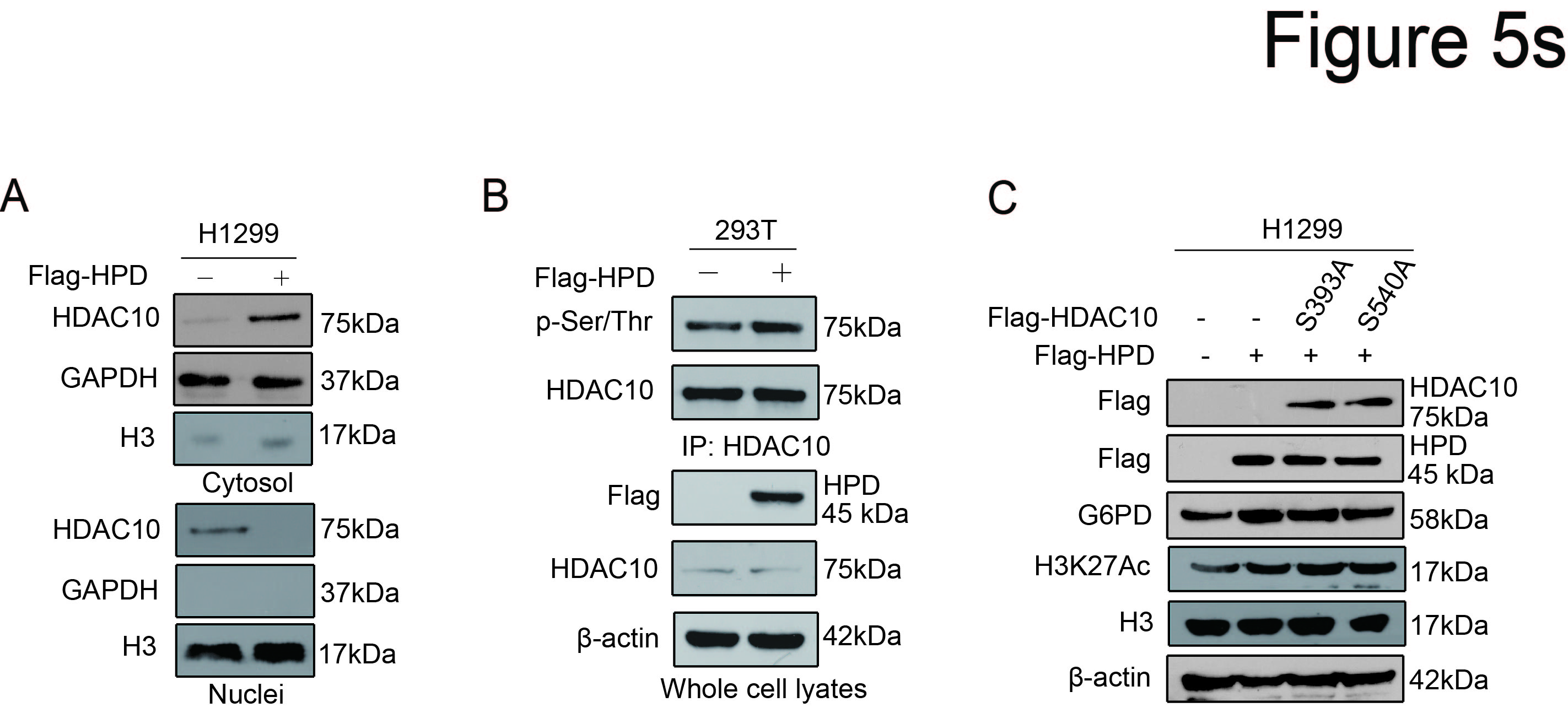

Supplement: Supplementary file 6 — Supplemental Figure 5 [file 41419_2019_1756_MOESM6_ESM.jpg]

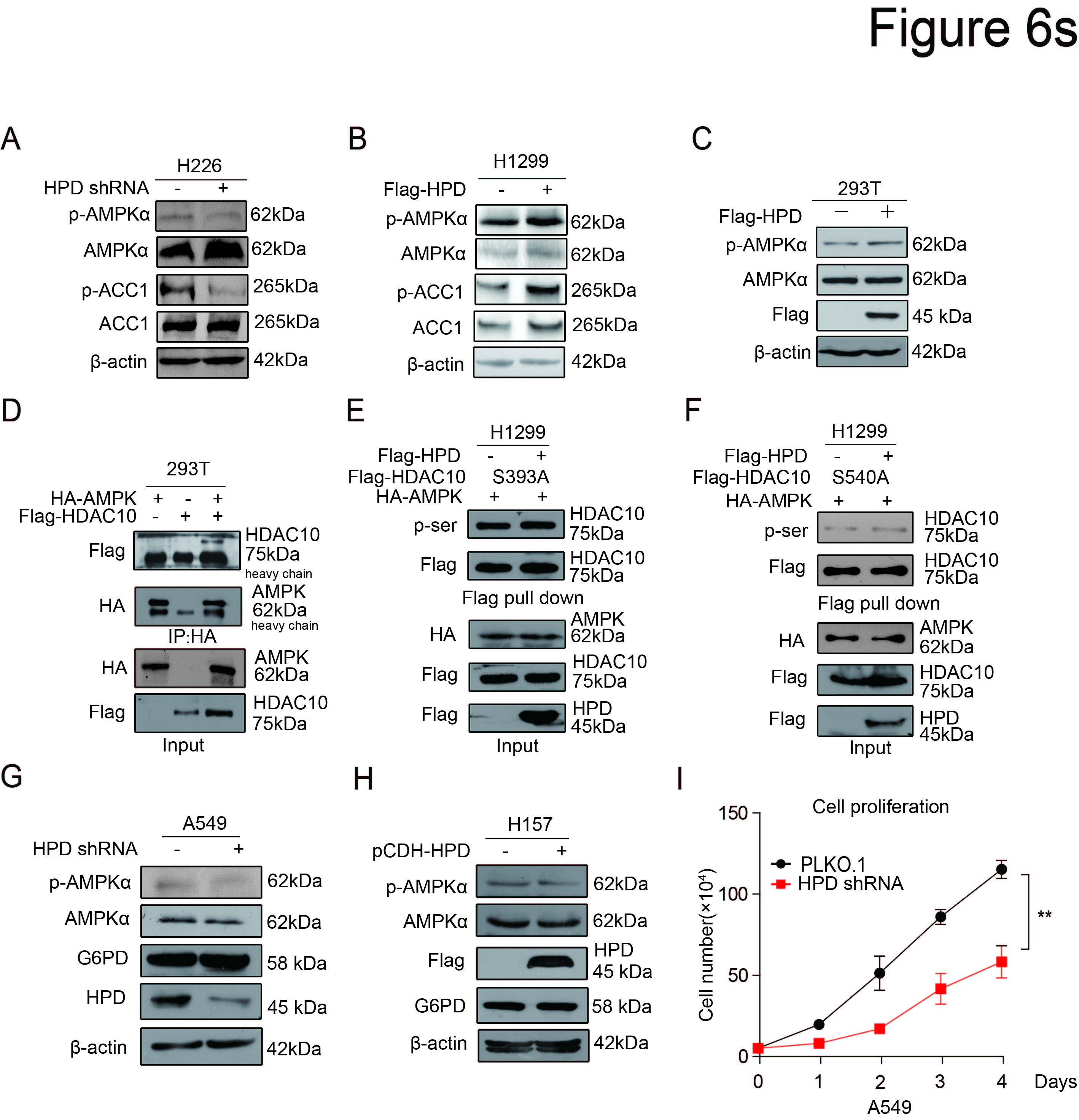

Supplement: Supplementary file 7 — Supplemental Figure 6 [file 41419_2019_1756_MOESM7_ESM.jpg]
