# Supplementary material for: 4-hydroxyphenylpyruvate dioxygenase promotes lung cancer growth via pentose phosphate pathway (PPP) flux mediated by LKB1-AMPK/HDAC10/G6PD axis
Source: Cell Death Dis. 2019 Jul 8;10(7):525. doi: 10.1038/s41419-019-1756-1 (PMC6614486; doi:10.1038/s41419-019-1756-1)
Supplement: Supplementary file 8 — Supplementary Table 1 [file 41419_2019_1756_MOESM8_ESM.pdf]

Supplementary Table 1. Expression of HPD protein in non-small cell lung cancer (NSCLC)

**Diagnosis**	**No.of case**	**HPD**				**Positive cases rate (%)**	**strong positive cases rate(%)**
		**-**	**+**	**++**	**+++**		
NSCLC	48	8	10	18	12	83.3%**	**62.5%****
Adjacent normal lung	48	37	6	5	0	22.9%	10.4%

Positive rate: percentage of positive cases with +, ++, and +++ staining score.

Strongly positive rate (high-level expression): percentage of positive cases with ++ and +++ staining score.

** *p*<0.01 compared with adjacent normal lung.
