# Supplementary material for: 4-hydroxyphenylpyruvate dioxygenase promotes lung cancer growth via pentose phosphate pathway (PPP) flux mediated by LKB1-AMPK/HDAC10/G6PD axis
Source: Cell Death Dis. 2019 Jul 8;10(7):525. doi: 10.1038/s41419-019-1756-1 (PMC6614486; doi:10.1038/s41419-019-1756-1)
Supplement: Supplementary file 9 — Supplementary Table 2 [file 41419_2019_1756_MOESM9_ESM.pdf]

Supplementary Table 2. Relationship between HPD protein overexpression and the clinicopathological features of NSCLC

Variables	No. of case (*n*)	HPD strong positive rate (%)	χ^2	*P* value
**Gender**				
Male	32	20(62.5%)	0.931	0.333
Female	16	10(62.5%)		
**Age (years)**				
>62	31	19(61.3%)	0.261	0.167
≤62	17	11(64.7%)		
**Tumor size**				
≤4 cm	21	13(61.9%)	0.483	0.667
＞4cm	27	17(63.0%)		
**Histological grade**				
Grade-1	12	4(33.3%)	0.808	0.044*
Grade-2	14	1178.6%)		
Grade-3	22	15(68.2%)		
**TNM stage**				
Ⅰ~Ⅱ	19	10(52.6%)	0.743	0.257
Ⅲ~Ⅳ	29	20(68.9%)		
**Lymph node status**				
N0	18	8(44.4%)	0.821	0.179
N+	30	22(73.3%)		

* $p<0.05$
