# Supplementary material for: 4-hydroxyphenylpyruvate dioxygenase promotes lung cancer growth via pentose phosphate pathway (PPP) flux mediated by LKB1-AMPK/HDAC10/G6PD axis
Source: Cell Death Dis. 2019 Jul 8;10(7):525. doi: 10.1038/s41419-019-1756-1 (PMC6614486; doi:10.1038/s41419-019-1756-1)
Supplement: Supplementary file 10 — Supplementary Table 3 [file 41419_2019_1756_MOESM10_ESM.pdf]

Supplementary Table 3. The sequence of PCR primers

Gene	Sequence
β-actin	Forward primer：5′- CATCGAGAAATTGAGACGGTG-3′
	Reverse primer：5′-CCTTGGAAGATGGTCTTGAT -3′
G6PD	Forward primer：5- TGAGTCAGACAGGCTGGAAC -3′
	Reverse primer：5′- CACGGAAAAGAGAGGAGATG-3′
6PGD	Forward primer：5′- ACGTGGACATCCGCAAAG -3′
	Reverse primer：5′- GACTCGTCATACTCCTGCTTG -3′
HPD	Forward primer：5′- GAGCAAGACAAGTTTGGGAAGG -3′
	Reverse primer：5′- GCATTTGGGCAGTTTAGGAA -3′
HDAC10	Forward primer：5′- CGCCATCTACTTCCACCCGA -3′
	Reverse primer：5′- CCTGTGTAGCCCGTGTTTCT -3′
