# Supplementary material for: 4-hydroxyphenylpyruvate dioxygenase promotes lung cancer growth via pentose phosphate pathway (PPP) flux mediated by LKB1-AMPK/HDAC10/G6PD axis
Source: Cell Death Dis. 2019 Jul 8;10(7):525. doi: 10.1038/s41419-019-1756-1 (PMC6614486; doi:10.1038/s41419-019-1756-1)
Supplement: Supplementary file 11 — Supplementary Table 4 [file 41419_2019_1756_MOESM11_ESM.pdf]

Supplementary Table 4. The sequence of HDAC10 WT, mutants, HPD and G6PD promoter primers

Gene	Sequence
HDAC10 WT	Forward primer：5′- GCTCTAGAGCCACCATGGACTACAAGGA CGATGACGACAAGGGTTCTGGAGGCACGACTTACAGTGACA AAG-3′<br>Reverse primer：5′- CGGGATCCTTACATGCCGGGCACCACCCC -3′
HDAC10 S368A	Forward primer：5′- GTGACCGCTGTGCCGATGGCCCC-3′<br>Reverse primer：5′- GAGTGGCTGCTGGGGCTCGCCG-3′
HDAC10 S373A	Forward primer 5′- AGCCCCAGCAGCCACGCCCCAGAGGG-3′<br>Reverse primer：5′- CGTGGCTGCTGGGGCTCATCGGCACA-3′
HDAC10 S393A	Forward primer：5′- TGTAAGGCAGCTGCAGCTGCACCGAG-3′<br>Reverse primer：5′- CTGCAGCTGCCTTACACACTGGACCC-3′
HDAC10 S540A	Forward primer：5′- GAGGCGGCTGCCCTAGCCATGTTCCA-3′<br>Reverse primer：5′- CTAGGGCAGCCGCCTCCTTGCCCCTG-3′
pCDH-Flag HPD	Forward primer: 5′-GCTCTAGAGCCACCATGGACTACAAGGA CGATGACGACAAGGGTTCTGGAGGCACGACTTACAGTGACA AAG-3′<br>Reverse primer: 5′- CGGGATCCTTACATGCCGGGCACCACCCC -3′
pcDNA3.1-Flag HPD	Forward primer: 5-CGGGATCCGCCACCATGGACTACAAGGACG ATGACGACAAGGGTTCTGGAGGCACGACTTACAGTGACAAA G-3′<br>Reverse primer: 5′-GCTCTAGATTACATGCCGGGCACCACCCC -3′
G6PD promoter	Forward primer：5′- CGGGGTACCCAATTGTTGAAGTTG GTAGC TGTGA -3′<br>Reverse primer：5′- CCGCTCGAGAAGTGTACGACCGTTT CC -3′
