# Supplementary figures and images for: Correction: 4-hydroxyphenylpyruvate dioxygenase promotes lung cancer growth via pentose phosphate pathway (PPP) flux mediated by LKB1-AMPK/HDAC10/G6PD axis
Source: Cell Death Dis. 2025 Feb 26;16(1):133. doi: 10.1038/s41419-025-07459-6 (PMC11865556; doi:10.1038/s41419-025-07459-6)

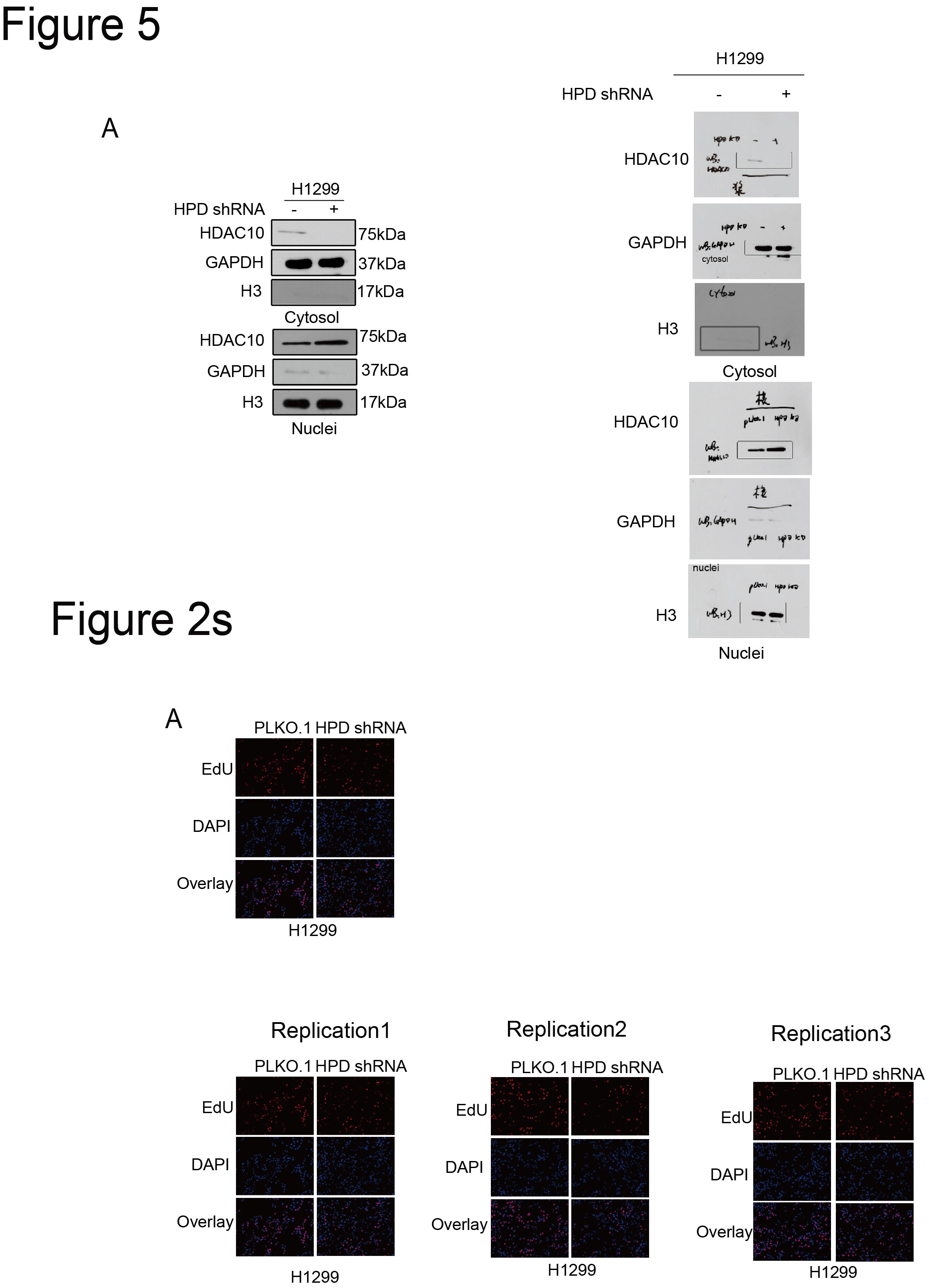

Supplement: Supplementary file 1 — Original data V1 [file 41419_2025_7459_MOESM1_ESM.jpg]

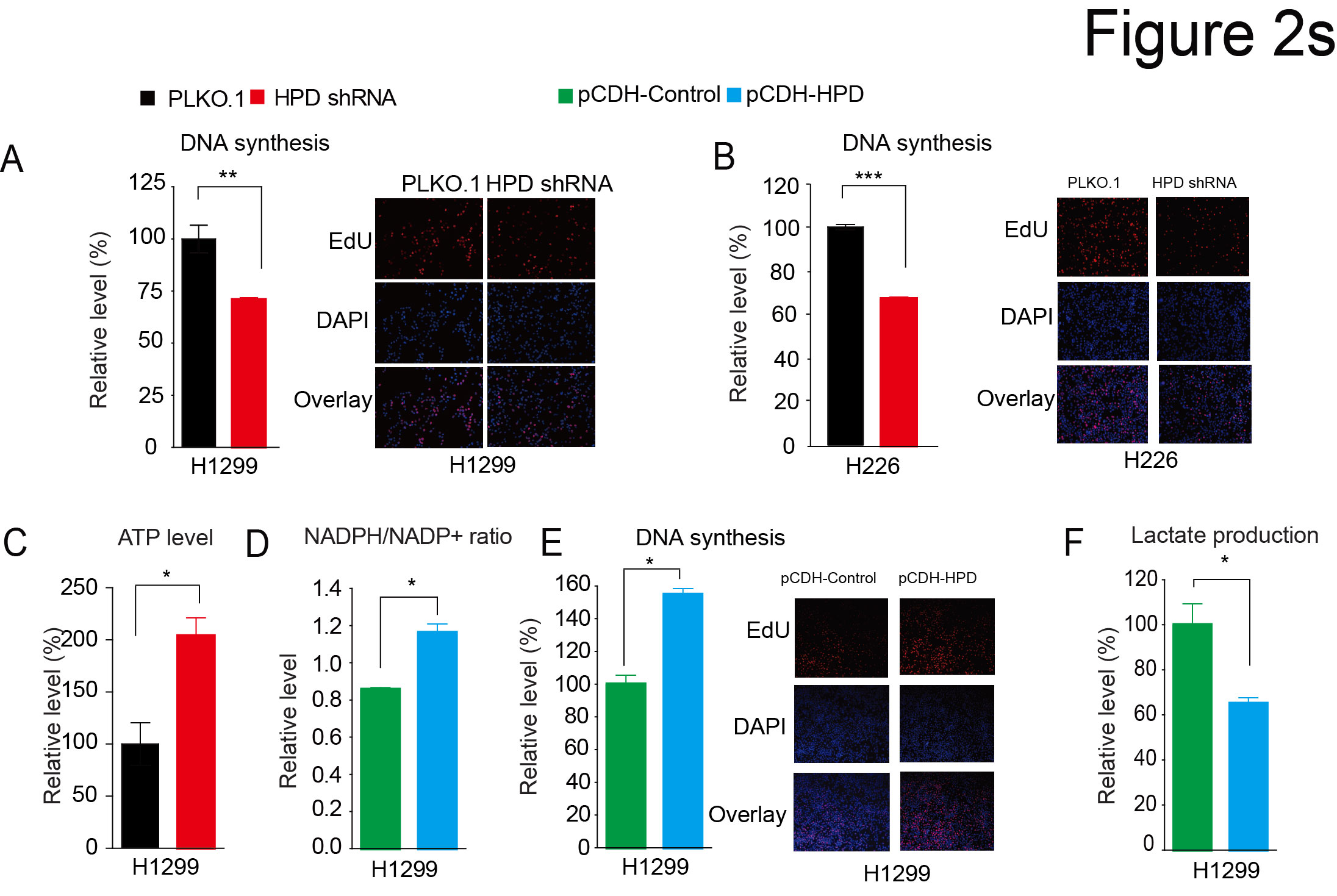

Supplement: Supplementary file 2 — Supplemental Figure 2 [file 41419_2025_7459_MOESM2_ESM.jpg]
